# Attachment Efficiency of Nanomaterials to Algae as an Important Criterion for Ecotoxicity and Grouping

**DOI:** 10.3390/nano10061021

**Published:** 2020-05-27

**Authors:** Kerstin Hund-Rinke, Tim Sinram, Karsten Schlich, Carmen Nickel, Hanna Paula Dickehut, Matthias Schmidt, Dana Kühnel

**Affiliations:** 1 Fraunhofer Institute for Molecular Biology and Applied Ecology, Auf dem Aberg 1, 57392 Schmallenberg, Germany; t.sinram@googlemail.com (T.S.); karsten.schlich@ime.fraunhofer.de (K.S.); 2 Institute for Energy and Environmental Technology, e.V. (IUTA), Bliersheimer Straße 58-60, 47229 Duisburg, Germany; nickel@iuta.de; 3 Helmholtz Centre for Environmental Research—UFZ, Permoserstr. 15, 04318 Leipzig, Germany; Hanna.P.D@gmx.de (H.P.D.); matthias.schmidt@ufz.de (M.S.); dana.kuehnel@ufz.de (D.K.)

**Keywords:** nanotoxicology, European Chemicals Agency (ECHA), ecotoxicology, nanoparticles, aggregation, *Raphidocelis subcapitata*

## Abstract

Engineered nanomaterials (ENMs) based on CeO_2_ and TiO_2_ differ in their effects on the unicellular green alga *Raphidocelis subcapitata* but these effects do not reflect the physicochemical parameters that characterize such materials in water and other test media. To determine whether interactions with algae can predict the ecotoxicity of ENMs, we studied the attachment of model compounds (three subtypes of CeO_2_ and five subtypes of TiO_2_) to algal cells by light microscopy and electron microscopy. We correlated our observations with EC_50_ values determined in growth inhibition assays carried out according to the Organisation for Economic Co-operation and Development (OECD) test guideline 201. Light microscopy revealed distinct patterns of ENM attachment to algal cells according to the type of compound, with stronger interactions leading to greater toxicity. This was confirmed by electron microscopy, which allowed the quantitative assessment of particle attachment. Our results indicate that algal extracellular polymeric substances play an important role in the attachment of ENMs, influencing the formation of agglomerates. The attachment parameters in short-term tests predicted the toxicity of CeO_2_ and TiO_2_ ENMs and can be considered as a valuable tool for the identification of sets of similar nanoforms as requested by the European Chemicals Agency in the context of grouping and read-across.

## 1. Introduction

Engineered nanomaterials (ENMs) show great variation in size, shape, crystalline structure, and surface modifications. According to the European Chemicals Agency (ECHA), grouping and read-across approaches can be applied to reduce the number of tests required for the risk assessment of ENMs [1]. ENM groups with analogous sets of physicochemical properties enable reasonable hazard predictions without additional testing, thus saving time and costs. Most concepts for the prediction of ENM properties focus on toxicity in humans [2,3]. Insight into ecotoxicity and grouping has been gained in systematic studies that generated ecotoxicological data for seven chemical species (Ag, ZnO, TiO_2_, CeO_2_, Cu, Fe, and SiO_2_) with 25 modifications [4,5]. Given the focus on regulatory applications, ecotoxicity was based on the Organisation for Economic Co-operation and Development (OECD) test guidelines 201 (algae), 202 (daphnids), and 236 (fish embryos). The studies considered reactivity, ion release, and morphology as properties indicating ecotoxicity. Nevertheless, it was difficult to separate subtypes of the same chemical species with this grouping approach. The ecotoxicity of TiO_2_ and CeO_2_ showed particularly broad ranges of subtype-dependent EC_50_ values (TiO_2_ = 0.2–126.9 mg/L, CeO_2_ = 8.5–99 mg/L). This suggests that additional parameters are needed to improve grouping, such as the adsorption of ENMs to algae [5]. The attachment of nanomaterials to green algae has already been reported [6,7,8,9] but these studies have not systematically addressed the attachment of different subtypes of the same ENM (and the relationship with ecotoxicity) or the quantity of ENMs attached to the algal cells.

We therefore investigated the attachment of ENMs to algae in order to determine whether this parameter can improve the results of ecotoxicological grouping. We focused on ENMs based on three subtypes of CeO_2_ and five subtypes of TiO_2_ differing in their ecotoxicological impact on algae, representing a subset of nanomaterials that have been comprehensively tested for aquatic and terrestrial ecotoxicity [4,5]. We studied the interaction between the ENMs and algal cells by light microscopy and quantified their behavior by scanning electron microscopy (SEM) with energy-dispersive X-ray spectroscopy (EDX).

## 2. Materials and Methods 

### 2.1. Selection of Nanomaterials

The eight ENMs (three subtypes of CeO_2_ and five subtypes of TiO_2_) have been characterized in detail, and their physicochemical properties (and the corresponding analytical methods) are described in the supporting information of two publications [4,5]. The ENMs considered herein originated mainly from the program “Testing a Representative set of Manufactured Nanomaterials” initiated by the OECD Working Party on Manufactured Nanomaterials [10,11]. Selected characteristics of the ENMs are listed in Table 1. Each ENM was used at a concentration of 100 mg/L to determine the agglomerate size, zeta-potential, and reactivity.

### 2.2. Preparation of Suspensions

The ENM suspensions were prepared as previously described [4]. Briefly, a stock suspension of each ENM (1 mg/mL) was prepared in ultrapure water by sonicating for 10 min using a cup horn (Bandelin, Germany) with a final energy input of 0.6 W/mL. A specific amount of the stock suspension was then applied to the test medium to achieve the target concentration for subsequent tests.

### 2.3. Algal Growth Inhibition Test

Growth of the green alga *Raphidocelis subcapitata* was measured as set out in the OECD test guideline 201 [12]. The growth rate was calculated by measuring chlorophyll fluorescence in vitro [15], with four replicates per test concentration and eight replicates for the control. Five to six test concentrations with a spacing factor of 2–3 were prepared. Furthermore, growth inhibition was compared in high-ionic-strength Grimme–Broadman (GB) medium and OECD medium (Table 2). 

### 2.4. Microscopy

Particle attachment to algae was observed by light and electron microscopy, the former for the rapid and inexpensive screening of the ENMs and the latter for more detailed tests at the single-cell level. Image evaluation was then used to estimate the coverage of cells by ENM particles, but this was labor-intensive and only a few individual cells could be analyzed per sample, reducing the statistical power. Image analysis was also unable to account for particles attached underneath the cells. The advantages and disadvantages of each method are summarized in Table 3.

#### 2.4.1. Attachment of ENMs to Algae by Light Microscopy

We carried out a short-term test and a growth inhibition test, and in each case observed the algae by light microscopy to investigate their interactions with the ENMs. For the short-term test, an algal culture was incubated in OECD medium (Table 2) until the cell density reached 3–4 million cells/mL. We then transferred 90 mL of this culture to a clean, sterile 250-mL Schott Duran Erlenmeyer flask and added 10 mL of the ENM stock dispersion to achieve a final concentration of 100 mg/L. The flask was then incubated for 3 h under the same conditions as the algal growth inhibition tests before removing samples for analysis. In addition, 3-h spike tests were carried out with fixed concentrations of NM-212 (100 mg/L) and varying algal cell densities (3,000,000, 1,400,000, 700,000 and 175,000 cells/mL). For microscopic analysis during the growth test, algae incubated with selected ENM concentrations were analyzed at the test end. 

We pipetted 20–50 µL of algal culture (growth tests and spike tests) onto a clean microscope slide and placed a cover glass on top. The slide was air dried at room temperature until the liquid under the cover glass had partially evaporated. We then sealed the edges to prevent further drying (which would cause the cells to shrivel and prevent detailed observation). To avoid the influence of physical or chemical parameters on the attached nanoparticles, we avoided reagents and protocols that might affect the structural integrity of the sample (e.g., paraformaldehyde or heat fixation). The algae were then observed using a Leica Primo Star (Leica Microsystems, Wetzlar, Germany) equipped with three Plan Achromat oil immersion objectives (10×, 40×, and 100×) as well as filters for phase-contrast microscopy. Only objects in the aqueous phase were considered. All images were captured using an AxioCam Erc 5s with an additional 10× lens (Carl Zeiss, Jena, Germany) for final magnifications of 100×, 400×, and 1000×. 

#### 2.4.2. Attachment of ENMs to Algae by Electron Microscopy (SEM-EDX)

The algal cells were exposed to 0.1 or 1 mg/L CeO_2_ NM-212 for 24 h in OECD or GB medium as described above for the growth inhibition test. For each concentration, we collected 5–6 samples of 10 mL. Each algal–ENM suspension was centrifuged for 5 min at 4400× *g* in a Heraeus Megafuge (Heraeus Institute, Hanau, Germany). The supernatant was discarded, and the pellet gently resuspended in 1 mL 4% paraformaldehyde in sodium cacodylate buffer, pH 7.4 (Electron Microscopy Sciences, Hatfield, PA, USA) for 15 h at 4 °C. The cells were then deposited onto polycarbonate filters (0.2 µm pore size) and washed for 15 min in sodium cacodylate buffer, before gradual dehydration (transfer to 30% ethanol in MilliQ water followed by the dropwise addition of 96% ethanol over 30 min to increase the final ethanol concentration to 93%). The filters were then transferred to absolute ethanol and critical point dried using a Leica EM CPD 300 device. In preparation for SEM, the samples were sputter-coated with a 30-nm gold–palladium (90/10) layer using a Leica EM SCD 500 instrument and mounted onto SEM stubs.

Samples were observed under a Zeiss Merlin VP Compact field-emitting SEM (Carl Zeiss Microscopy, Oberkochen, Germany) with a Bruker ((Bruker Nano Analytics, Berlin, Germany)) QUANTAX FlatQUAD X-ray spectrometer. The electron acceleration voltage was set to 10 kV and the beam current to ~300 pA, which achieved the ionization of cerium while allowing for cell surface imaging with an Everhard-Thornley secondary electron detector. Cerium maps were obtained from spatially resolved EDX data using the Ce L-alpha line.

The coverage of algal cells with ENMs was determined from overlays of SEM and EDX images generated using the Correlia Plugin for ImageJ/Fiji [17,18]. Based on these overlays, Fiji tools were used to calculate the area of each image covered with algae and the area covered with cerium as well as algae. From these values, we were able to calculate the coverage as a percentage. In each of the 5–6 samples collected from the two different media (GB and OCED) and the two different ENM concentrations (0.1 and 1 mg/L), we analyzed 2–7 individual cells by EDX in order to determine the mean coverage. 

### 2.5. Statistical Evaluation

Statistical analysis and the calculation of EC_50_ values in the algal growth inhibition tests were carried out using ToxRat Professional v3.3 (ToxRat Solutions, Alsdorf, Germany). Dose-response functions were determined by linear regression (probit model) and EC_50_ confidence limits were based on Fieller’s theorem. 

## 3. Results

### 3.1. Attachment of CeO_2_

#### 3.1.1. Light Microscopy

**Figure 1:**
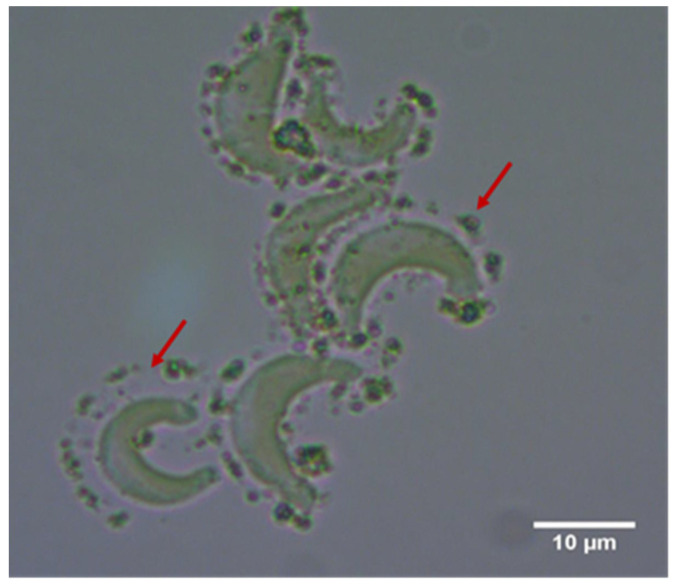
Nanoparticle attachment to transparent, sheath-like structures around algal cells (red arrows). This phase-contrast image (1000× magnification) was captured after 3 h incubation with 100 mg/L CeO_2_ NM-212.

**Figure 2:**
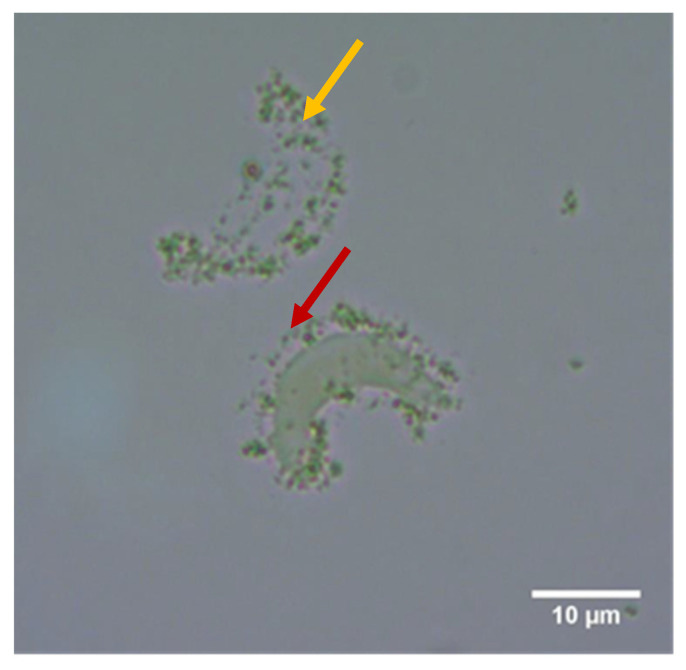
Nanoparticle attachment to transparent, algal-shaped structures with (red arrow) and without (yellow arrow) a corresponding algal cell. This phase-contrast image (1000× magnification) was captured after 3 h incubation with 100 mg/L CeO_2_ NM-212.

CeO_2_ NM-212 showed strong attachment to algal cells during spike experiments after a contact period of 3 h (Figure 1 and Figure 2). There was mostly no direct contact with the cell wall, but instead the ENMs attached to a transparent sheath around the individual algal cells. We also observed the attachment of NM-212 to a transparent structure, with a shape similar to the algal cells. The formation of agglomerates was highly dependent on the concentration ratio between the algal cells and nanoparticles. The 3-h spike experiments showed that small agglomerates (~0.1 mm) formed at the highest and lowest cell densities (3,000,000 and 175,000 cells/mL), whereas larger agglomerates (0.5–2 mm) formed at the intermediate cell densities (1,400,000 and 700,000 cells/mL). Although the appearance of the agglomerates was dependent on cell density, the attachment of ENMs to individual algal cells was similar across all spike experiments. Even algal cells embedded within thicker and larger agglomerates featured the same gap between the cell surface and attached nanoparticles. These transparent structures were not observed in the controls without NM-212, indicating they were induced by the presence of the ENM.

The attachment of ENMs to the algal cells was observed in the algal growth inhibition tests at all test concentrations after 72 h. As described for the spike experiments, the size of the agglomerates was highly dependent on the concentration ratio between the algal cells and nanoparticles (Figures S1–S3). The size of agglomerates, as determined by microscopy, initially increased in line with the ENM test concentration (2.5–10 mg/L) but decreased again at the highest value (40 mg/L). The distinct shell-like attachment, which we identified during spike experiments, was less pronounced during the growth inhibition tests perhaps due to the lower ENM concentrations. CeO_2_ NM-211 showed attachment behavior comparable to CeO_2_ NM-212 (Figure S4).

**Figure 3:**
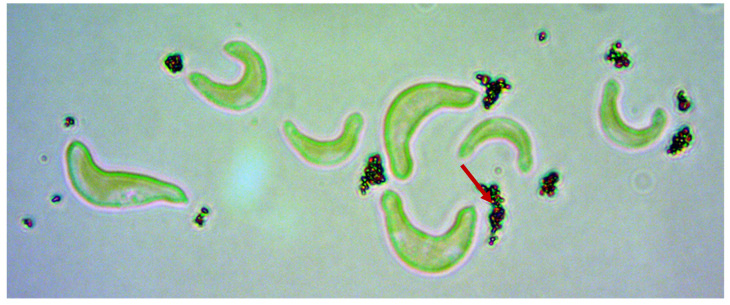
Nanoparticle attachment to transparent, sheath-like structures around algal cells (red arrow). This phase-contrast image (1000× magnification) was captured after 3 h incubation with 100 mg/L CeO_2_ NM-213.

**Figure 4:**
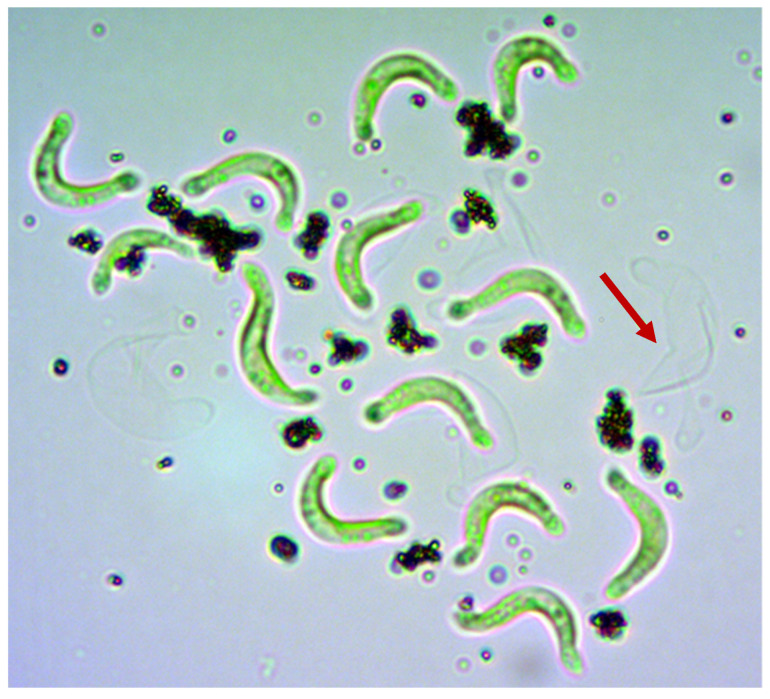
Transparent, algae-shaped structures (red arrow). This phase-contrast image (1000× magnification) was captured after 3 h incubation with 100 mg/L CeO_2_ NM-213.

In contrast, CeO_2_ NM-213 showed much weaker attachment to *R. subcapitata* cells during both the spike experiments and algal growth inhibition tests. Despite this weak interaction, a transparent sheath-like structure was again observed around the algal cells, preventing direct contact between the ENM and cell surface (Figure 3). Transparent, algae-shaped structures were also observed with few or no particles attached (Figure 4).

As described for the spike experiments, only a few particles attached to the algal cells during the growth inhibition tests after 72 h (Figures S5–S8). Accordingly, the shell-like structures around the algal cells were less obvious, although many transparent algae-shaped structures featuring a small number of attached particles were observed following the test period, especially at the higher test concentrations.

#### 3.1.2. SEM-EDX Analysis

**Figure 5:**
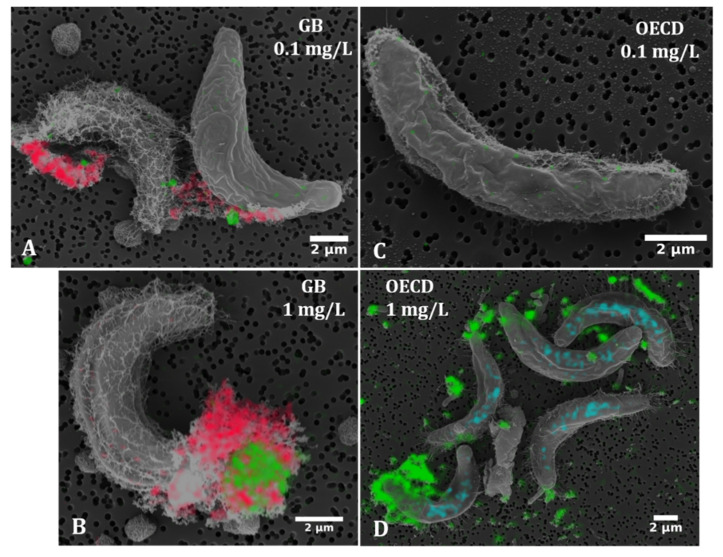
SEM-EDX (energy-dispersive X-ray spectroscopy) images of *Raphidocelis subcapitata* cells following exposure to CeO_2_ NM-212 at two concentrations in two media, GB (**A**,**B**) and OECD (**C**,**D**). Some cells appear to be covered by network-like structures (potentially extracellular polymeric substances or artifacts that appear during sample preparation). Optically identical particulate assemblies are associated with the cells and, based on EDX analysis, contain not only cerium (green), but also sodium (blue) and iron (red).

We studied the attachment of CeO_2_ NM-212 to individual *R. subcapitata* cells by electron microscopy. Given the observed differences in toxicity (Section 3.3), we also compared particle attachment following incubation in two different media: GB and OECD. In both cases, we observed the attachment of CeO_2_ NM-212 particles to algal cells and this was confirmed by EDX (Figure 5). Control cells that were not exposed to the ENM are shown in Figure S9. Interestingly, EDX analysis showed that not all particles associated with the algae were composed of cerium, but that iron and sodium were also present. The iron particles were deposited on the algae exclusively when the cells were incubated in GB medium and given that no iron-containing chemicals were used during sample preparation we conclude that iron in the GB medium precipitated onto the cells. Sodium precipitation was occasionally observed in the samples incubated in OECD medium. Furthermore, most cells were surrounded by filamentous web-like envelopes, probably the shrunken remains of extracellular polymeric structures (EPS) visible by light microscopy. Fewer of these structures were associated with the control cells (Figure S9). These network-like structures could also be artifacts generated during sample preparation.

**Figure 6:**
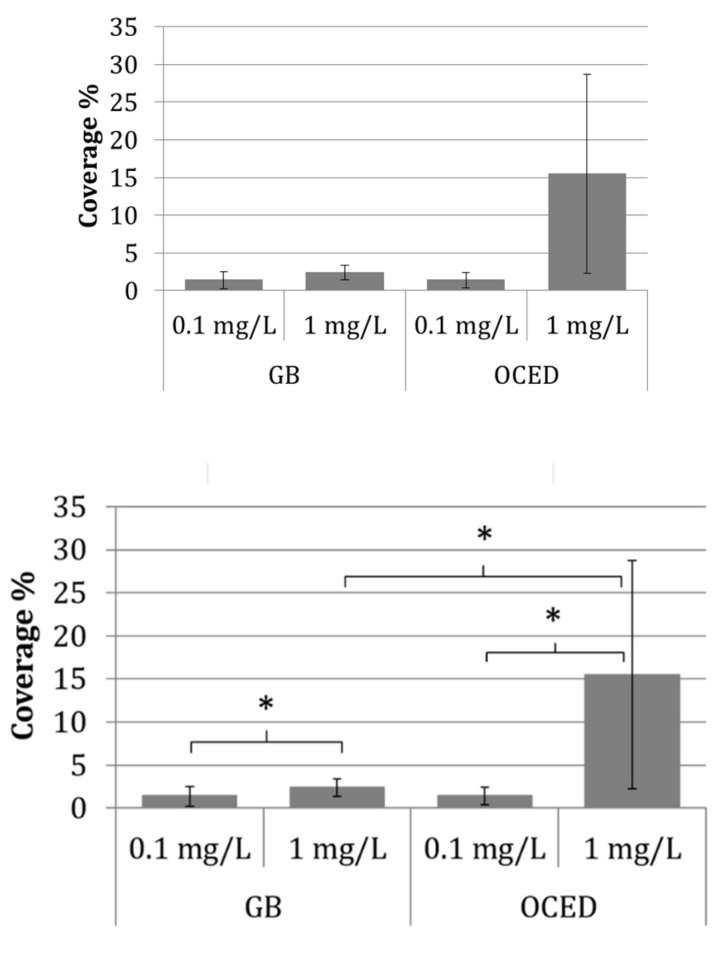
Coverage of the algal surface with cerium from NM-212 particles as determined by SEM-EDX image overlay analysis. We analyzed 5–6 samples per medium (GB and OECD), each sample comprised 2–7 cells. The data are means ± standard deviations. One-tailed unpaired *t*-test (Microsoft Excel 2013) revealed significant differences (marked by *) between the concentrations (*p* = 0.033 in GB medium, *p* = 0.044 in OECD medium) as well as between the media at 1 mg/L (*p* = 0.045).

The coverage of algal cells with NM-212 particles was quantified from the overlay of SEM and EDX images. This showed no significant difference between the GB and OECD media at an ENM concentration of 0.1 mg/L (2–3% coverage in each case), but a trend towards higher coverage in OECD medium (14% in OECD vs. 2.4% in GB) at an ENM concentration of 1 mg/L (Figure 6). 

### 3.2. Attachment of TiO_2_

The analysis of TiO_2_ ENMs by light microscopy revealed that, like CeO_2_ NM-211 and NM-212, the TiO_2_ particles formed agglomerates that attached to the algal cells (Figures S10–S20). However, these particles formed heterogeneous agglomerates that differed in terms of compactness and the manner of attachment. Specifically, they primarily formed shell-like single layers of compact agglomerates around the algal cells but also formed loose agglomerations of cells and particles. Like the non-doped TiO_2_ particles (Figure S10), the Eu-doped TiO_2_ particles densely covered the algal cells and formed compact agglomerates up to 1 mm in diameter, as well as loose agglomerations with less-ordered attachments (Figures S11 and S12). In contrast, the Fe-doped TiO_2_ particles formed only shell-like single layers of compact agglomerates (Figure S13). The gap between the TiO_2_ NM-105 particles and algal cells was wider and the shell-like structure was less dense compared to the doped particles (Figure S14). NM-104 particles attached to the algae sparingly and formed a fragmented rather than a complete compact shell, and the width of the gap varied from indistinguishable up to a clearly defined space (Figure S15). 

Microscopic analysis during the growth inhibition tests revealed the formation of a shell-like layer for the highest test concentration of the Eu-doped TiO_2_ particles (18 mg/L) and large but loose agglomerates at a lower concentration (2 mg/L) near the EC_50_ value (Figures S16 and S17). In contrast, the NM-104 agglomerates formed loose and open structures at the lowest test concentration of 7.5 mg/L (Figures S18–S20). Although most algal cells were incorporated into agglomerates, the status of individual cells was dependent on the ENM concentration, with few if any surface particles at the lowest test concentration (7.5 mg/L) or at 30 mg/L, but all algal cells incorporated into agglomerates at the highest test concentration of 120 mg/L.

### 3.3. Growth Inhibition Tests

For all three CeO_2_ ENMs and two of the five TiO_2_ ENMs, we carried out growth tests based on OECD test guideline 201 [12] and compared our results to earlier experiments [5]. We also compared the toxicity of CeO_2_ NM-212, Eu-doped TiO_2_, and non-doped TiO_2_ in the two media and found that all three ENMs were more toxic in OECD medium than GB medium (Table 4). 

We compared the results generated by the two laboratories involved in this study (Fraunhofer Institute for Molecular Biology and Applied Ecology and Helmholtz Centre for Environmental Research) and also compared our results to published data [5]. In most cases, there was less than a five-fold variation. The ecotoxicity of the three CeO_2_ ENMs differed by a factor of 100, with NM-213 showing the lowest toxicity. The ecotoxicity of the TiO_2_ ENMs also differed by a factor of 100, with the non-doped and Eu-doped ENMs showing the highest toxicity and NM-104 the lowest.

### 3.4. Relationship between Attachment Behavior and Ecotoxicity

**Figure 7:**
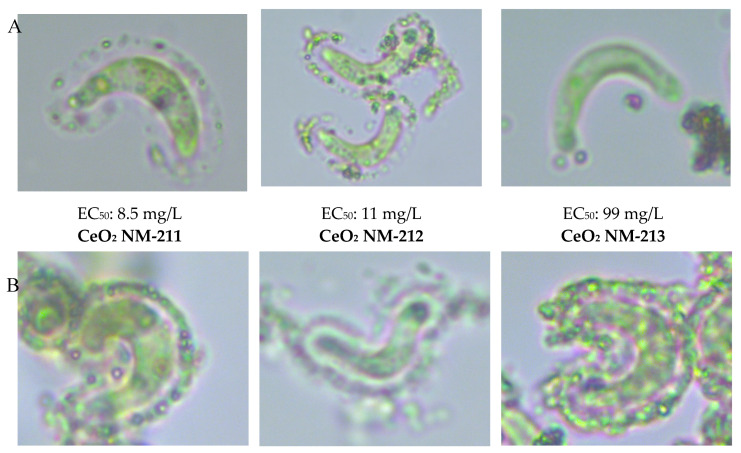

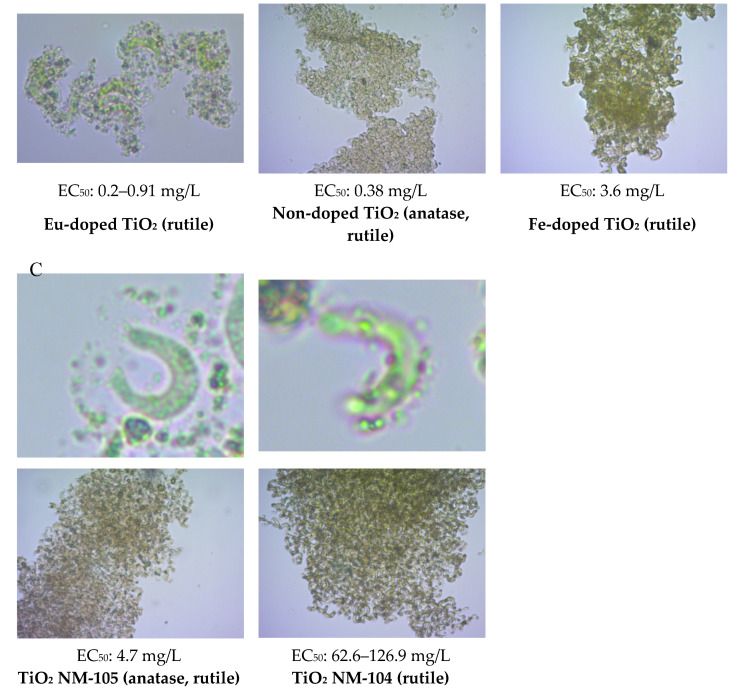
Comparative analysis of the EC_50_ values and attachment behavior of three CeO_2_ ENMs (**A**) and five TiO_2_ ENMs (**B**,**C**) (magnification 100× and 1000×).

We observed a clear relationship between ecotoxicity and attachment efficiency for the three CeO_2_ ENMs (Figure 7). NM-211 and NM-212 were highly toxic and also showed a great propensity for attachment to algal cells, whereas NM-213 was much less toxic and formed few agglomerations. In contrast, the relationship between ecotoxicity and attachment was more complex for the TiO_2_ ENMs (Figure 7). There was no clear division between the particles that favored and disfavored interactions with algae, but rather a gradual change from strong to weak attachment. The Fe-doped ENM showed the strongest attachment, forming shell-like structures in compact agglomerates with most algal cells, followed by the Eu-doped and non-doped ENMs (mostly shell-like structures in compact agglomerates but some looser agglomerates), NM-105 (loose shell-like structures, gaps between algae and particles), and finally NM-104 (shell-like fragments, large gaps between algae and particles). The Eu-doped and non-doped ENMs showed the highest toxicity, followed by the Fe-doped ENM and NM-105, and finally NM-104. Furthermore, there was no obvious relationship between the crystalline structure of TiO_2_ (rutile or rutile/anatase) and ecotoxicity.

## 4. Discussion

### 4.1. Attachment Behavior

Different attachment characteristics were observed depending on the type of ENM interacting with the algal cells. The ENMs in the spiking experiment did not attach directly to the algal surface but to a transparent, peripheral structure. Algae can excrete EPS [19] and increase the production of these molecules when under osmotic stress [20,21] or chemical stress caused by toxic chemicals [22,23,24,25,26] or natural toxin exudates [27]. Depending on their ability to bind the algal cell wall, EPS are generally categorized as soluble or bound substances [26,28]. They comprise many different organic acids, amino acids, peptides, sugars, polysaccharides, and oligosaccharides [20,28,29], and their composition varies among different species [30]. The role of EPS in the agglomeration of cells and ENMs has been reported before [31]. Their protective function has been demonstrated while investigating the toxicity of Ag nanoparticles toward the alga *Chlorella pyrenoidosa*, where EPS-extracted cells were more sensitive to the nanoparticles than control cells [29]. EPS production depends on the chemical substance that induces stress. For example, three ENMs (TiO_2_, SiO_2_, and CeO_2_) were tested for their effect on the green alga *Dunaliella tertiolecta*, and although the EPS response in all cases was regulated by the Ca^2+^ signaling pathway, SiO_2_ induced a 200–800% increase in EPS production whereas TiO_2_ had only a limited effect [32]. A given ENM can also have different effects on EPS production in different algae: for example, antifouling agents induce a stronger EPS response in *Scenedesmus* sp. than *Chlorella* sp., mirroring the toxicity profiles against these species [33].

Existing test guidelines developed for chemical substances have been reevaluated to determine their suitability for ENMs, and any necessary adaptations have been identified. Furthermore, new test guidelines are under development and guidance documents have been provided [34]. One of the recommendations was a modified growth test with green algae [15,35]. We therefore considered modifications such as the use of fluorescence to determine algal cell numbers. The results of our growth tests using the same ENM under identical test conditions at different times and in different laboratories varied in most cases by less than a factor of five, confirming the robustness of the method and justifying its use to compare ecotoxicological data with attachment behavior (Table 4). Our results also varied (in most cases) by less than a factor of five compared to earlier experiments [5]. This is lower than the factor of 10 which is considered adequate for the definition of ecotoxicity categories for pesticides [36]. We also observed a clear relationship between the ecotoxicity of CeO_2_ in the growth inhibition test and the attachment of ENMs to algae in the spiking experiment. The ENMs induced a shell-like structure in the short-term test, which was also observed in the growth test with NM-213. Such a structure was not observed in the control groups without ENMs, indicating that CeO_2_ ENMs trigger two effects: first they induce the formation of a shell-like structure (presumably EPS) to protect the algae; and second, the nanoparticles adsorb to the EPS (depending on the type of ENM) which correlates with the toxicity in the growth test. 

The interaction between ENMs and EPS is complex, probably reflecting the stressor-dependent composition of the EPS. For example, the infrared spectrum of the EPS induced by Ag differed from that induced by TiO_2_ in the same algal species [33]. The adsorption of ENMs to algae is also influenced by surface modifications, as shown by the relatively stronger interactions between Ag nanoparticles and algae when the ENM was coated with polyvinylpyrrolidone rather than citrate [29]. Furthermore, P25 (14% rutile and 86% anatase) and anatase ENMs show higher affinity for proteins and polysaccharides whereas rutile ENMs attach more efficiently to phospholipids [37]. Anatase TiO_2_ adsorbs more efficiently to bound EPS than rutile TiO_2_, which may explain its greater ecotoxicity [38].

NM-105 has an anatase and rutile crystalline structure specifically designed for photocatalysis, and the remarkably wide gap between algae and the shell-like structure formed in the presence of NM-105 indicates a thick EPS layer, presumably induced by the increased production of reactive oxygen species. The combination of loose attachment (compared to the Fe-doped ENM) and the high reactivity (due to its photocatalytic activity) could explain the ecotoxicity of NM-105, which was comparable to the doped TiO_2_ ENMs. The relationship between EPS induction and the toxicity of TiO_2_ and Ag was reported previously [33] and the underlying causes of the interaction between EPS and metal(loid)s have been comprehensively reviewed [28].

The evidence from our study and previous reports therefore suggests that attachment behavior can be used as a surrogate parameter to indicate ecotoxicity. The attachment of ENMs to algal cells is merely an observation of interaction behavior, and although our ENMs were characterized in detail, the physicochemical properties responsible for the different attachment behaviors could not be identified. There is no obvious direct relationship between attachment behavior and parameters such as primary particle size, surface area, morphology, surface chemistry, crystalline structure, zeta-potential, isoelectric point, agglomeration size, reactivity, solubility or ecotoxicity [4,5]. 

The growth of the algae in our experiments was inhibited by the ENMs, indicating a toxic effect despite the production of protective extracellular substances [28]. The toxicity of Ag ENMs may reflect the penetration of the cell wall and plasma membrane followed by the intracellular release of Ag^+^ [29]. Similarly, TiO_2_ ENMs may penetrate the cell and inhibit the synthesis of chlorophyll *b* and carotenoids [9]. However, TiO_2_ ENMs also promote significant hydrophobic interactions by forming complexes of titanium acetate between the aliphatic –COOH groups of EPS and titanium ions [38]. This involves the significant accumulation of TiO_2_ ENMs on the EPS but almost no penetration, yet toxic effects were nevertheless observed (particularly for the anatase ENM compared to the rutile ENM, corresponding to the extent of adsorption) [38]. The mode of action is unclear but may involve the reduced penetration of light caused by the shell of adsorbed nanoparticles. Shading due to sorption is considered a nanospecific effect in contrast to shading by turbid test dispersions [35]. Toxicity could also reflect the increased energy demand due to the production of EPS, thus reducing the growth rate. 

A spike experiment addressing attachment and agglomeration behavior can only be considered as a proxy for ecotoxicity. We found that the agglomeration behavior depends on the concentration ratio between the algal cells and ENM, but in the spike experiment the concentration of both components was high. Furthermore, the EC_50_ values of the ENM vary by several orders of magnitude, indicating that the concentration ratio changes during the 72-h growth test as the algal cell number increases while the ENM concentration stays the same. The high concentration of the algae and ENMs in the spike test therefore differs from the conditions in the growth inhibition test. The stronger induction of EPS production may explain the different behavior of the toxic NM-212 particles in the growth inhibition and spike tests, whereas the discrepancy was minimal for the less toxic NM-213 particles. However, the relationship between attachment and ecotoxicity is plausible, and spike experiments, despite their weaknesses, could therefore be useful to determine the ecotoxicity of ENMs toward algae. The ENM characterization and screening experiments revealed similar sizes for the agglomerates. For example, the agglomerate size for NM-212 was 830 ± 200 nm in the characterization experiments (Table 1) and ~1 µm in the screening assay (Figure 1). We used the same ENM concentration in both cases (100 mg/L in OECD medium). This confirms that no significant artifacts were introduced during the preparation of samples.

For NM-212, the quantification of surface coverage on the algal cells indicated a strong correlation between attachment and toxicity. In GB medium, the EC_50_ for the CeO_2_ particles was >100 mg/L and the coverage was ~2%, whereas in OECD medium the EC_50_ for the same material was 1.8 mg/L and the coverage was ~14%. The difference in toxicity may reflect the compositions of the two media, with the rich GB medium containing more components at higher concentrations hence providing better growth conditions (Table 2). The coverage is likely to be underestimated (while preserving the trend) because particle detachment may occur during sample preparation. The light microscopy data indicate higher coverage due to the higher concentrations of ENMs used in the spike assays (100 mg/L compared to 1 mg/L). However, the detection of sodium and iron precipitates on the cell surface indicates that particles remain attached during fixation. Sodium precipitates were detected on cells after incubation in OECD medium, which is rich in sodium (Table 2), whereas iron was detected in cells incubated in GB medium, which contains Fe-EDTA. The presence of medium-specific elements on the cell surface after fixation indicates that particle attachment is strong enough to endure multiple washing steps, although it is possible that fixation may have a differential effect on the elements iron, sodium, and cerium.

The electron microscopy data were inconclusive regarding the modulation of EPS formation by different ENMs in the two media. A network-like structure was clearly present on the surface of the algal cells, but there was no clear difference between cells exposed to different ENMs, between ENM-exposed cells and controls, or between cells incubated in GB and OCED media. The fixation procedure may be too harsh to preserve EPS structures in a state that allows their thickness to be compared across different treatment groups.

### 4.2. Read-Across and Grouping Strategies

In the read-across technique, endpoint information for one chemical is used to predict the same endpoint for another, which is considered similar in some way [39]. For nanomaterials, the approach is only permitted in the context of various forms of the same substance [1]. Additionally, grouping is an essential part of the ECHA draft guidance on the registration of sets of similar nanoforms: “…A ‘set of similar nanoforms’ is a group of nanoforms … where the clearly defined boundaries in the parameters … of the individual nanoforms within the set still allow to conclude that the hazard assessment … of these nanoforms can be performed jointly. A justification shall be provided to demonstrate that a variation within these boundaries does not affect the hazard assessment … of the similar nanoforms in the set” [40]. In the grouping and read-across concept, a nanoform is an ENM with the same crystalline structure, comparable particle size distribution, morphology, surface functionalization, and surface area [40]. Sufficient similarity can be assumed if there is preferably a logical ranking of the materials that allows the identification of a worst case [40]. 

Given the different attachment and agglomeration behaviors of the TiO_2_ and CeO_2_ ENMs, read-across may solely be possible between various forms of the same ENM. The ECHA does not define threshold values for physicochemical parameters, and the three CeO_2_ nanomaterials could therefore be assigned to two groups. However, different groupings arise from the selection of different physicochemical parameters: based on the primary particle size, NM-211 is smaller than NM-212 and NM-213; based on the surface area, NM-213 differs from NM-211 and NM-212; and in the algal ecotoxicity tests, NM-211 and NM-212 are much more toxic than NM-213. Therefore, grouping based on individual physicochemical parameters is considered to be less targeted. We still lack parameters with a clear relationship to the test organisms, but in algae the attachment behavior of the ENMs may provide an acceptable proxy. For the three CeO_2_ ENMs, there was a clear correlation between attachment behavior and ecotoxicity.

The selected physicochemical properties of TiO_2_ ENMs also affect the results of grouping. The five ENMs form one group based on size, but grouping by surface area leads to the separation of Eu-doped TiO_2_, grouping by reactivity leads to the separation of NM-105, and grouping by crystalline structure separates the rutile/anatase NM-105 and Fe-doped TiO_2_ from the three rutile ENMs. NM-104 can also form a separate group as the only coated ENM. If all these differences are considered at the same time, the five TiO_2_ ENMs serve as representatives of five groups. However, this is not justified for the assessment of ecotoxicity toward algae. Based on the empirical data, we have three groups differing in ecotoxicity by a factor of 10, but they have heterogeneous physicochemical properties. We observed comparable ecotoxicity for ENMs differing in crystalline structure and reactivity (NM-105 is anatase/rutile and reactive, whereas Fe-doped TiO_2_ is rutile and non-reactive) or differing in surface area (Eu-doped TiO_2_ = 148 m^2^/g, whereas non-doped TiO_2_ = 78 m^2^/g). As discussed for CeO_2_, attachment behavior appears to be a better indicator of similar ecotoxicity toward algae than physicochemical parameters. The materials with the strongest attachment behavior and lowest reactivity (Eu-doped and non-doped TiO_2_) showed the greatest ecotoxicity, followed by the material with the highest reactivity and moderate attachment efficiency (NM-105). The large gap between the ENMs and algal cells, indicating a shell-like structure for protection, can be explained by the high reactivity of the ENM. The material with low attachment efficiency and low reactivity (NM-104) showed the lowest ecotoxicity. Only the ecotoxicity of Fe-doped TiO_2_ cannot be explained by the attachment behavior and reactivity. A higher ecotoxicity than Eu-doped and non-doped TiO_2_ would be expected, and additional work is required to improve the prediction of the ecotoxicity for this material and corresponding ENMs. The underlying properties responsible for the different attachment behaviors are still unclear, but until these are identified it should be possible to use the easily-measured parameter of attachment behavior as a proxy for ecotoxicity, at least in the case of CeO_2_ and TiO_2_ ENMs. 

## 5. Conclusions

In this study, we used light microscopy and SEM-EDX to investigate the attachment of three CeO_2_ and five TiO_2_ ENMs to the green alga *R. subcapitata* and compared the attachment behavior with ecotoxicity data based on a growth inhibition assay following OECD test guideline 201. Light microscopy allowed us to screen the algae without compromising the structural integrity of the sample, whereas SEM-EDX provided images of greater resolution combined with elemental analysis. We found that CeO_2_ and TiO_2_ ENMs induce the formation of EPS by the algae and we observed a relationship between ecotoxicity (based on growth inhibition data) and the attachment behavior of the ENMs. In contrast, there was no simple relationship between algal ecotoxicity and the physicochemical properties of the ENMs. Attachment behavior can therefore be considered as a valuable proxy for the identification of sets of similar nanoforms in the context of grouping and read-across, which reduce the number of tests required for risk assessment. Our observations are based on the analysis of sparingly soluble, spherical ENMs available as white powder. Further experiments are required to determine whether attachment behavior has a similar predictive power for the ecotoxicity of different ENM shapes (such as rods, fibers, or platelets) and colors (such as red Fe_2_O_3_). Colored materials indicate the selective reflection of different wavelengths of light, so we cannot exclude potential interference with the pigments involved in photosynthesis and algal growth. Furthermore, ENMs that release ions such as Ag^+^ should also be tested.

## Figures and Tables

**Table 1:** Selected physicochemical characteristics of the eight engineered nanomaterials (ENMs) investigated in this study (for further details see the supporting information in two earlier publications [4,5]).

Nanomaterial	Primary Particle Size (SEM/TEM) (nm)	Surface Area (m^2^/g) ^1^	Agglomerate Size–Z Average (nm) (DLS) ^2^	Zeta-Potential (mV) ^2^	Reactivity (DMPO) ^2,3^	Crystalline Structure	Coating
CeO_2_ NM-211	4–15	66	442 ± 85	−19.8	0.81	Cubic cereonite	Uncoated
CeO_2_ NM-212	40	27	831 ± 209	−20.4	0.96	Cubic cereonite	Uncoated
CeO_2_ NM-213	35	4	1042 ± 178	−25.9	1.1	Cubic cereonite	Uncoated
TiO_2_ NM-104	30	60	1596 ± 498	−0.9	1.1 UV activation: 1.6	Rutile	Al_2_O_3_ (6%) coating and glycerol (1%) functionalization
TiO_2_ NM-105	21	51	1409 ± 533	−2.4	1.0 UV activation: 20.8	14% Rutile 86% anatase	Uncoated
TiO_2_ Eu-doped	19 (BET)	148	1612 ± 384	−23.1	0.7 UV activation: 1.4	Mainly rutile	Uncoated
TiO_2_ Fe-doped	10 (BET)	63	1866 ± 106	−21.3	1.0 UV activation: 1.4	Mainly rutile	Uncoated
TiO_2_ non-doped	15	78	743 ± 859	−22	0.7 UV activation: 1.5	9% Rutile, 91% anatase	Uncoated

^1^ Based on the BET (information provided by the manufacturers). ^2^ Determined in the Organisation for Economic Co-operation and Development (OECD) test medium [12] used for the growth test with algae. The pH was not adjusted but reached values between 7.0 and 7.4 (values >1.3 indicate reactivity). The values are presented as sample-to-blank ratios (*n* = 3). ^3^ Measurement of hydroxyl radicals generated after UV irradiation via Fenton-type reactions in the presence of hydrogen peroxide and 5,5-dimethyl-1-pyrroline-*N*-oxide (DMPO) [13,14]. SEM = scanning electron microscopy, TEM = transmission electron microscopy, DLS = dynamic light scattering, BET = Brunauer, Emmett and Teller specific surface area, UV = ultraviolet.

**Table 2:** Composition of Grimme–Broadman (GB) medium [16] and OECD medium [12].

	GB Medium (µmol/L)	OECD Medium (µmol/L)
KNO_3_	8000	–
NaCl	8000	–
MgSO_4_ * 7 H_2_O	1000	60.9
Na_2_HPO_4_ * 2 H_2_O	1000	–
NaH_2_PO_4_ * H_2_O	3000	–
CaCl_2_ * 2 H_2_O	100	122
MnCl_2_ * 4 H_2_O	2.5	2.1
H_3_BO_3_	8	2.99
ZnSO_4_ * 7 H_2_O	0.7	–
Na_2_MoO_4_ * 2 H_2_O	0.016	0.0289
FeEDTA ^1^	25	–
NH_4_Cl	–	280
KH_2_PO_4_	–	9.19
MgCl_2_ * 6 H_2_O	–	59
ZnCl_2_	–	0.022
CoCl_2_ * 6 H_2_O	–	0.0063
CuCl_2_ * 2 H_2_O	–	0.00006
Na_2_EDTA * 2 H_2_O	–	0.269
FeCl_3_ * 6 H_2_O	–	0.237
Ionic strength	24.349	1.602

^1^ Composed of FeSO_3_; * 7 H_2_O and Na_2_EDTA * 2 H_2_O.

**Table 3:** Advantages and disadvantages of light and electron microscopy for the observation of particle–cell interactions.

Light Microscopy	Electron Microscopy
Time and cost efficient	Time and cost intensive
No fixation and preparation	Fixation and preparation of samples may lead to artifacts (e.g., particles may be washed off)
Qualitative	Quantification of attachment possible
No identification of elements (may lead to artifacts)	Element identification ensures particle identity
Lower resolution compared to electron microscopy	Lower concentrations of particles and smaller particles / agglomerates can be detected

**Table 4:** EC_50_ values determined in growth tests (OECD medium) with the green alga *Raphidocelis subcapitata* ^1^.

Nanomaterials	EC_50_ (mg/L)	EC_50_ (mg/L) Published Data ^2^	EC_50_ (mg/L) ^5^ GB
CeO_2_-NM-211	Not performed	8.5 [7.7–9.3]	Not performed
CeO_2_ NM-212	10.9 [9.9–11.9] ^4^ 1.8 [n.d.] ^5^	5.6 [3.0–10.4]	>100
CeO_2_ NM-213	98.7 [96.5–101.4] ^4^	43.8 [n.d.] ^3^	Not performed
TiO_2_ NM-104	126.9 [95.0 ± 190.4] ^4^	62.6 [42.6–106]	Not performed
TiO_2_ NM-105	Not performed	4.7 [3.5–5.5]	Not performed
TiO_2_ Eu-doped	0.36 [0.34 ± 0.38] ^4^ 0.29 [n.d.] ^5^	0.91 [0.75–1.10]	>100
TiO_2_ Fe-doped	Not performed	3.6 [2.6–4.8]	Not performed
TiO_2_ Non-doped	0.06 [n.d.] ^5^	0.38 [0.33–0.43]	>100

^1^ Values in brackets = confidence interval. ^2^ Tests carried out as described in Section 2 and results presented in the supporting information of a previous study [5]. ^3^ n.d. = not determined. ^4^ Data from this study (Fraunhofer Institute for Molecular Biology and Applied Ecology). ^5^ Data from this study (Helmholtz Centre for Environmental Research).

## Supplementary Materials

The following are available online at https://www.mdpi.com/2079-4991/10/6/1021/s1.
