# Supplementary material for: Attachment Efficiency of Nanomaterials to Algae as an Important Criterion for Ecotoxicity and Grouping
Source: Nanomaterials (Basel). 2020 May 27;10(6):1021. doi: 10.3390/nano10061021 (PMC7352665; doi:10.3390/nano10061021)
Supplement: Supplementary file 1 [file nanomaterials-10-01021-s001.pdf]

# Supporting material

## Attachment efficiency of nanomaterials to algae as an important criterion for ecotoxicity and grouping

**Kerstin Hund-Rinke [1*], Tim Sinram [1], Karsten Schlich [1], Carmen Nickel [2], Hanna Paula Dickehut [3], Matthias Schmidt[3], Dana Kühnel [3]**

[1] Fraunhofer Institute for Molecular Biology and Applied Ecology; Auf dem Aberg 1, 57392 Schmallenberg, Germany

[2] Institute for Energy and Environmental Technology. V.(IUTA), Bliersheimer Straße 58-60, 47229 Duisburg, Germany

[3] Helmholtz Centre for Environmental Research (UFZ), Permoserstr. 15, 04318 Leipzig, Germany

* Correspondence: kerstin.hund-rinke@ime.fraunhofer.de

# 1 Attachment of $CeO_2$ NM-212 to the green algae *Raphidocelis subcapitata*

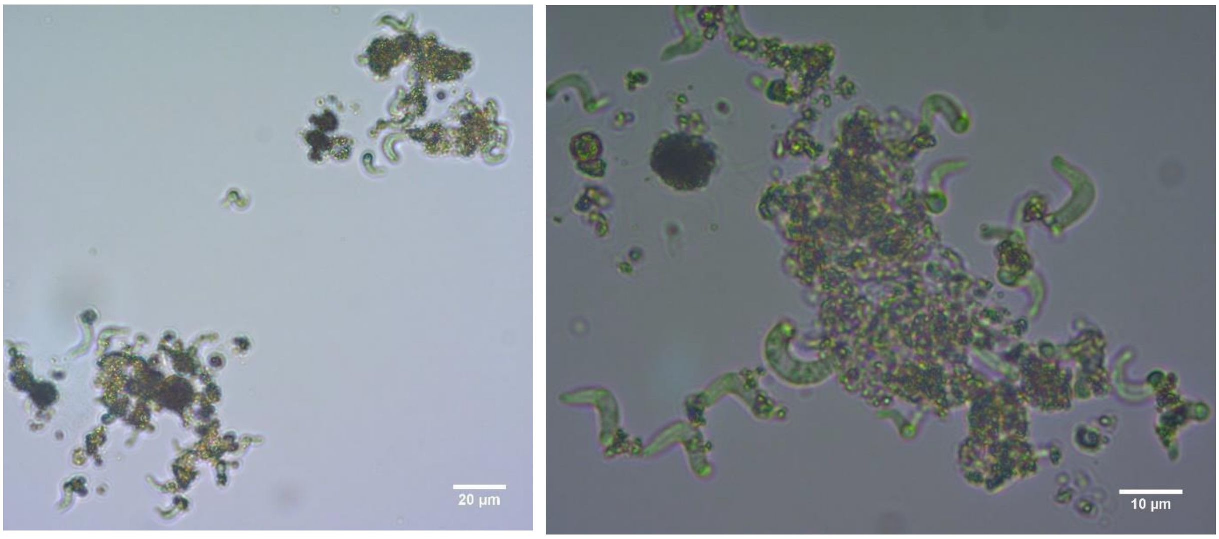


*Figure S 1: Attachment of $CeO_2$ NM-212 to algae. This phase-contrast image (left = 400×, right = 1000×) was captured during a 72-h growth inhibition test with 2.5 mg/L $CeO_2$ NM-212.*

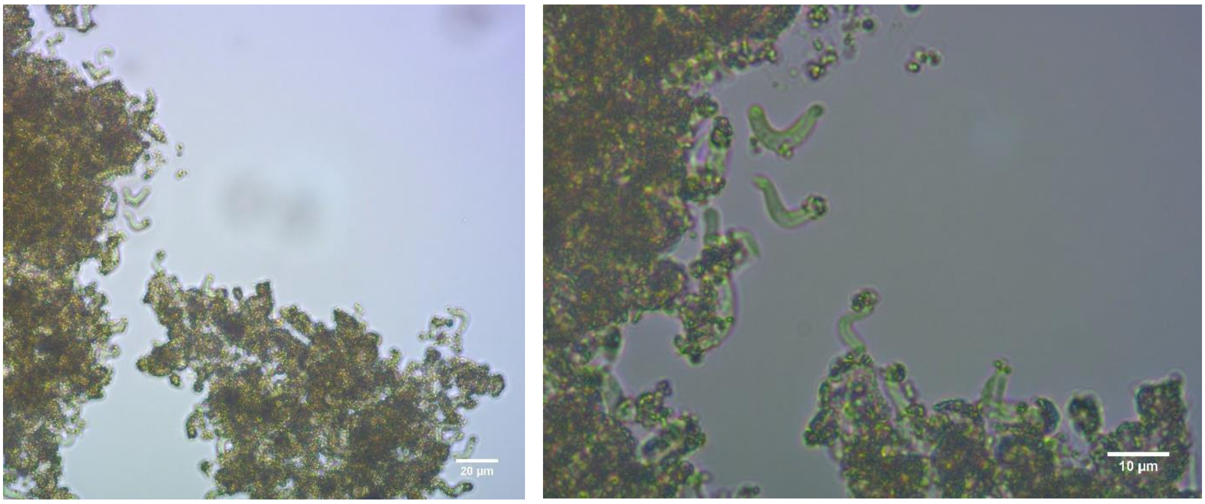


*Figure S2: Attachment of $CeO_2$ NM-212 to algae. This phase-contrast image (left = 400×, right = 1000×) was captured during a 72-h growth inhibition test with 10 mg/L $CeO_2$ NM-212.*

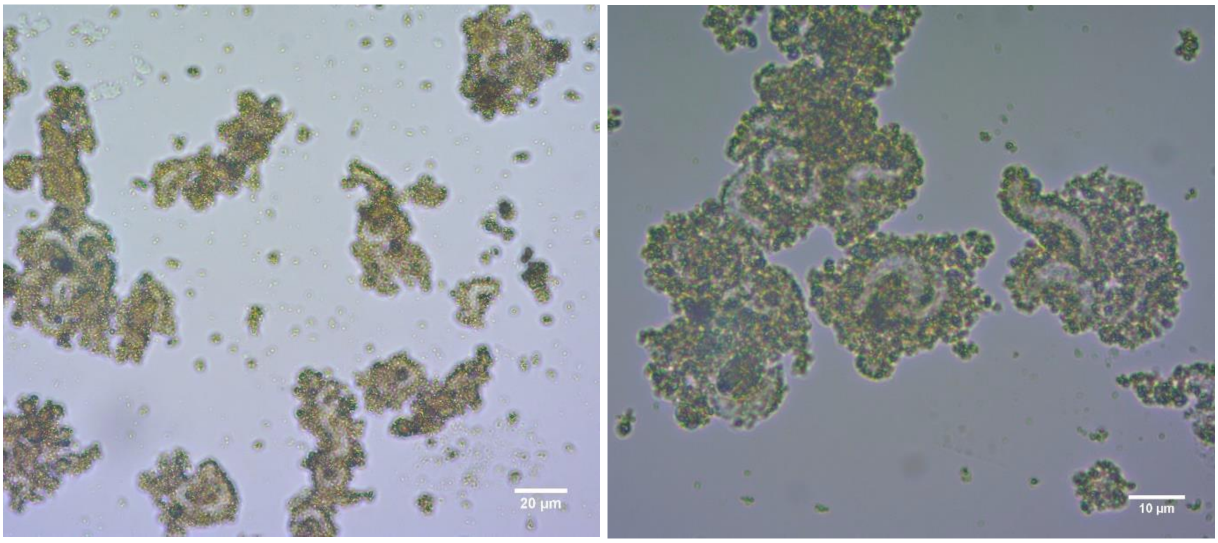


*Figure S3:* *Attachment of $CeO_2$ NM-212 to algae. This phase-contrast image (left = 400×, right = 1000×) was captured during a 72-h growth inhibition test with 40 mg/L $CeO_2$ NM-212.*

## 2 Attachment of $CeO_2$ NM-211 to the green algae *Raphidocelis subcapitata*

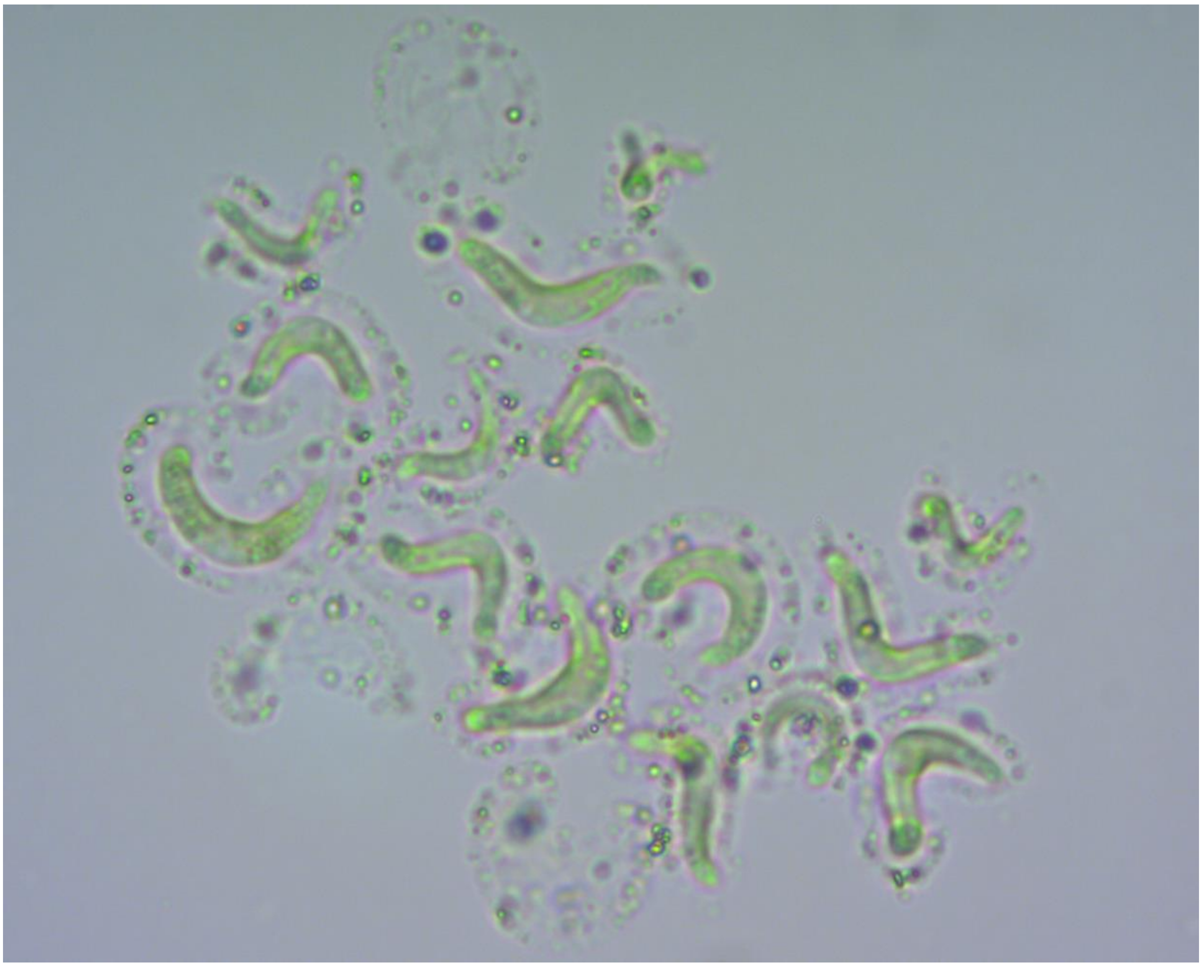

*Figure S4: Attachment of $CeO_2$ NM-211 to algae. This phase-contrast image (1000× magnification) was captured after 3 h incubation with 100 mg/L $CeO_2$ NM-211.*

# 3 Attachment of $CeO_2$ NM-213 to the green algae *Raphidocelis subcapitata*

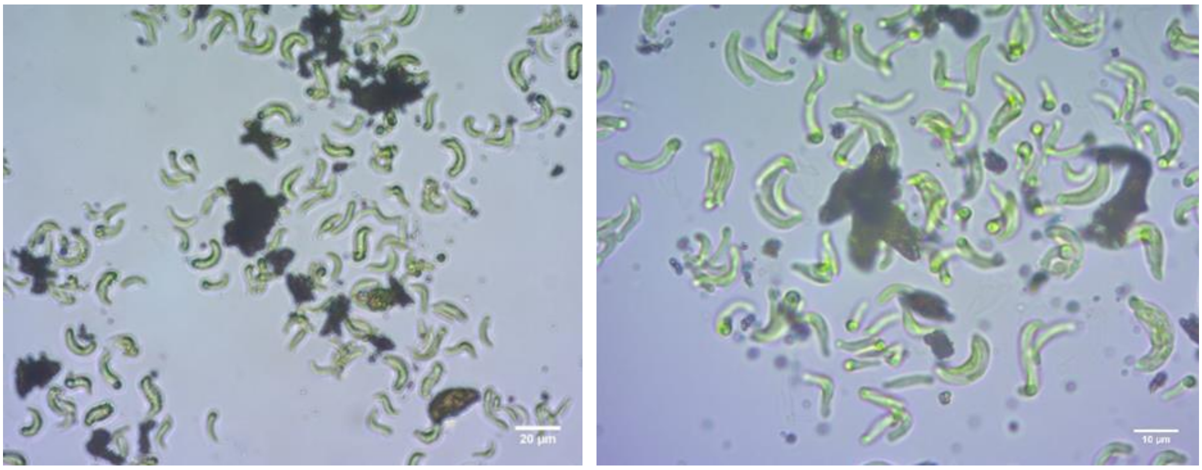


*Figure S5: Attachment of $CeO_2$ NM-213 to algae. This phase-contrast image (left = 400×, right = 1000×) was captured during a 72-h growth inhibition test with 10 mg/L $CeO_2$ NM-213.*

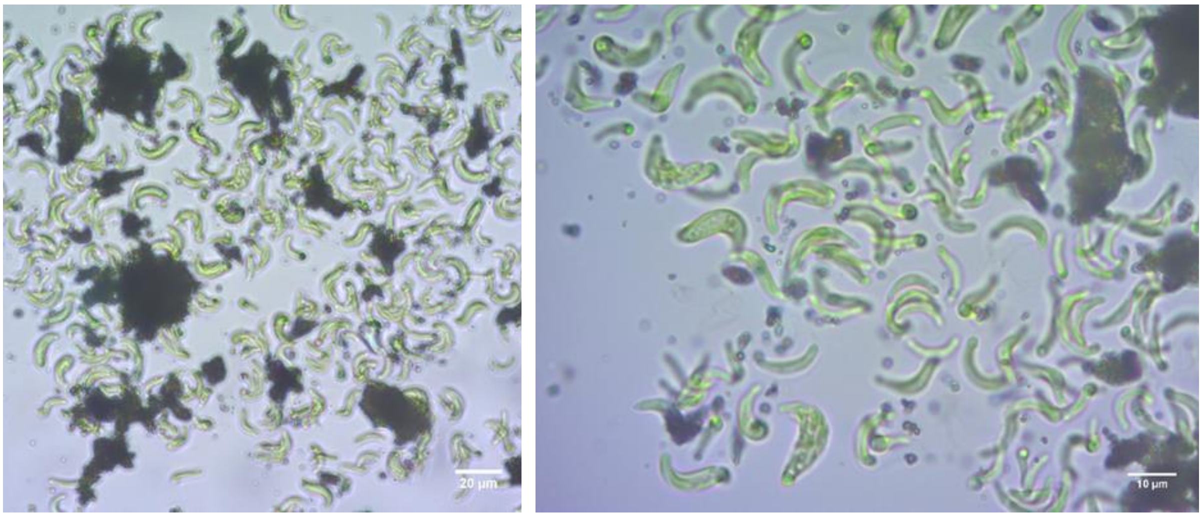


*Figure S6: Attachment of $CeO_2$ NM-213 to algae. This phase-contrast image (left = 400×, right = 1000×) was captured during a 72-h growth inhibition test with 24 mg/L $CeO_2$ NM-213.*

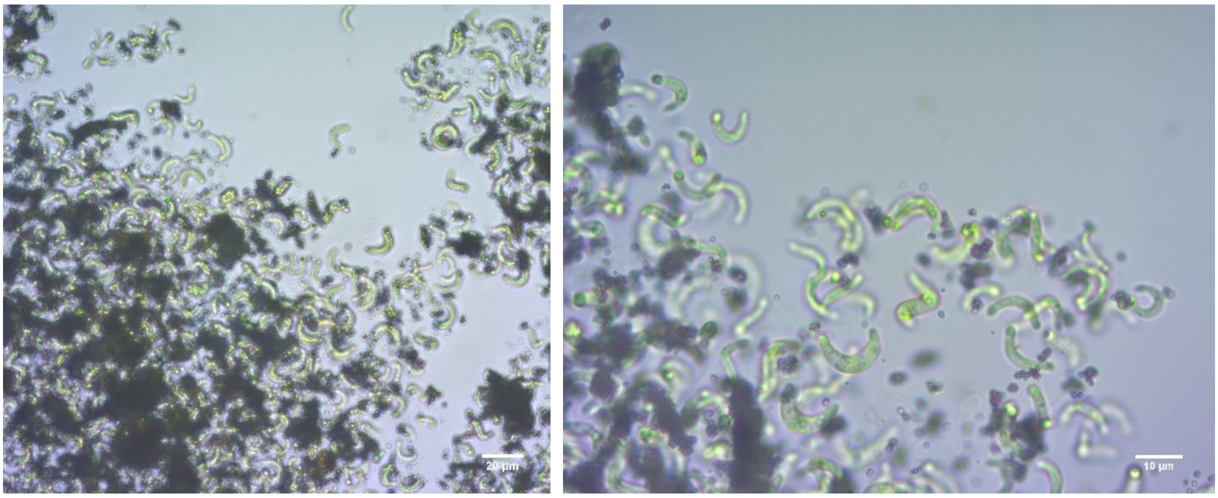


*Figure S7:* *Attachment of CeO2 NM-213 to algae. This phase-contrast image (left = 400×, right = 1000×) was captured during a 72-h growth inhibition test with 80 mg/L $CeO_2$ NM-213.*

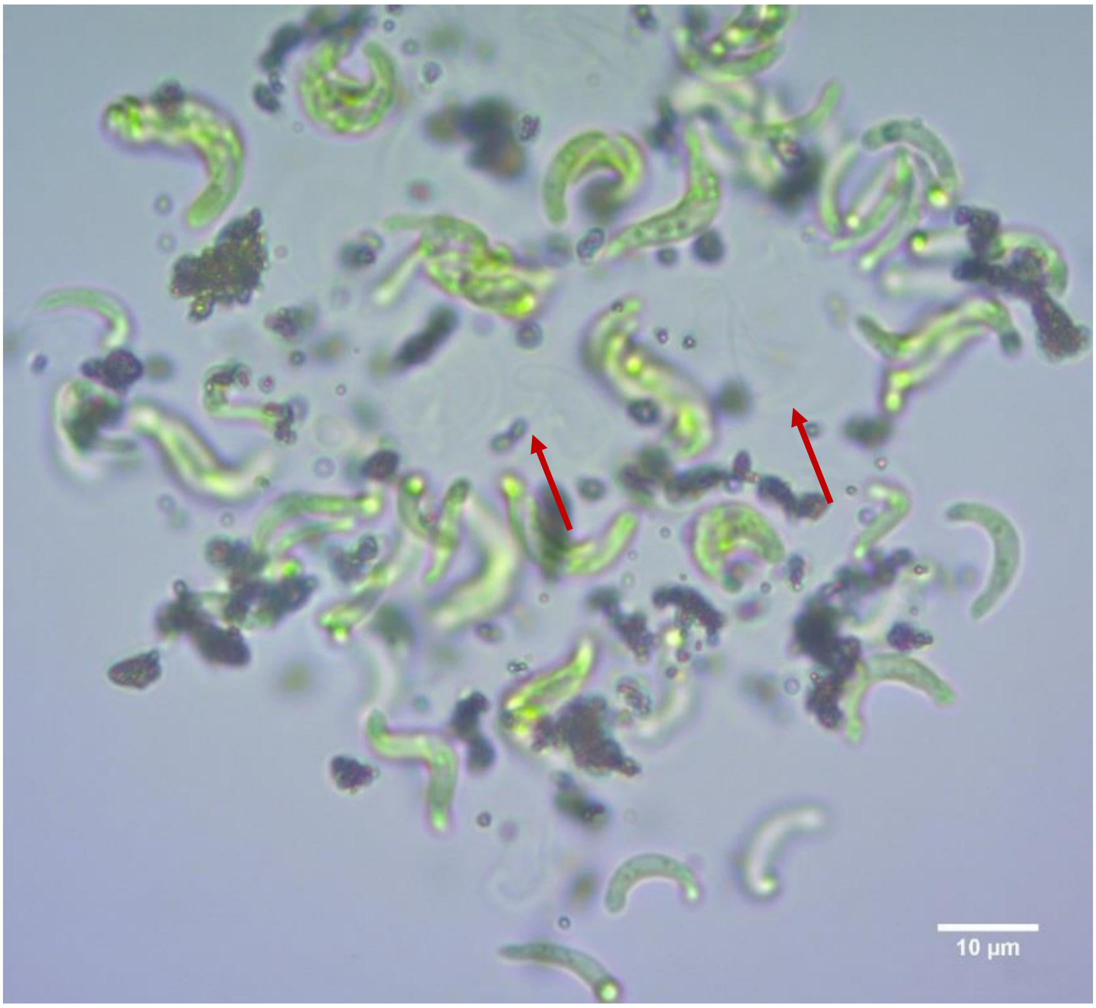


*Figure S8:* *Attachment of $CeO_2$ NM-213 to algae, showing transparent, algae-shaped structures (red arrow). This phase-contrast image (1000× magnification) was captured during a 72 h growth inhibition test with 80 mg/L $CeO_2$ NM-213.*

# 4 Control algae *Raphidocelis subcapitata*

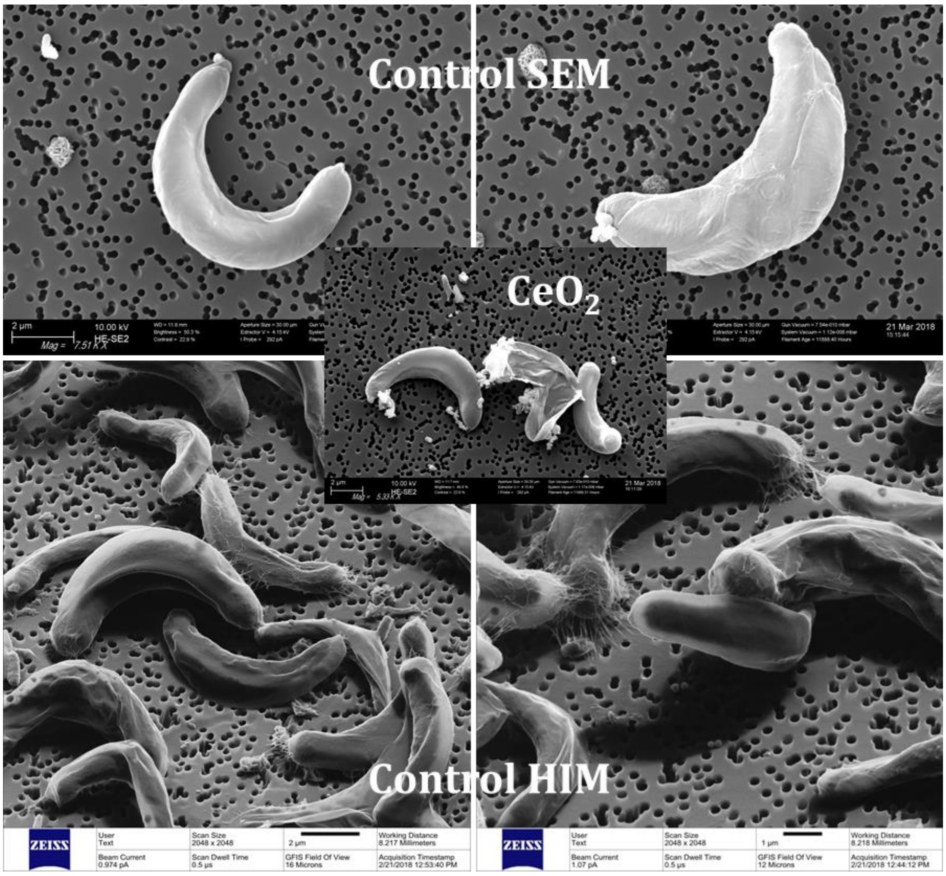


*Figure S9:* *Control algae Raphidocelis subcapitata. Upper panel: scanning electron microscopy (SEM) images. Lower panel: helium ion microscopy (HIM) images. Middle insert: SEM image of $CeO_2$ NM212 exposed algae cells.*

## 5 Attachment of $TiO_2$ non-doped (91 % anatase; 9 % rutile) to the green algae *Raphidocelis subcapitata* – short-term test

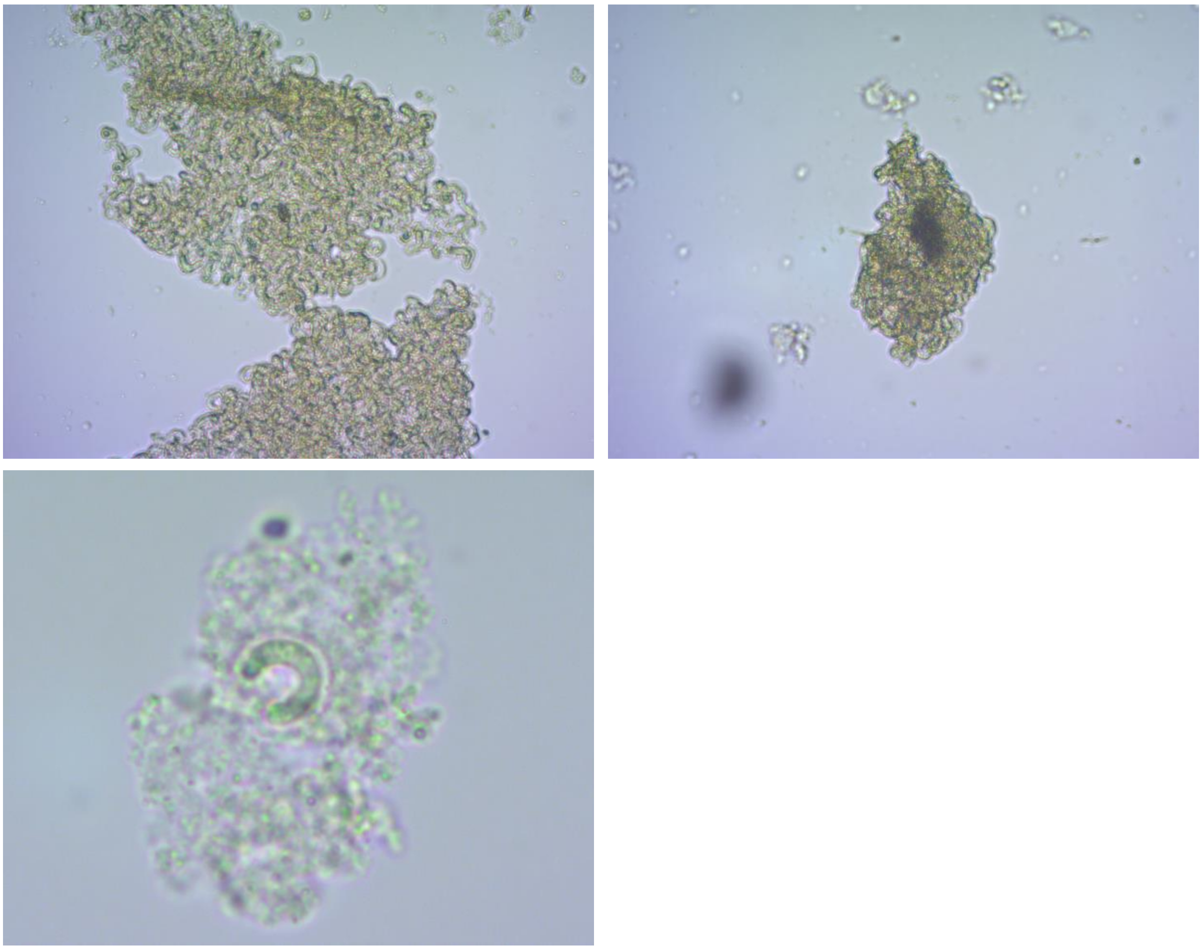

*Figure S10: Attachment of non-doped $TiO_2$ (91% anatase and 9% rutile) to algae, showing large agglomerates with embedded algal cells. This phase-contrast image (upper 400×, lower 1000×) was captured after 3 h incubation with 100 mg/L non-doped $TiO_2$.*

# 6 Attachment of Eu-doped $TiO_2$ to the green algae *Raphidocelis subcapitata* – short-term test

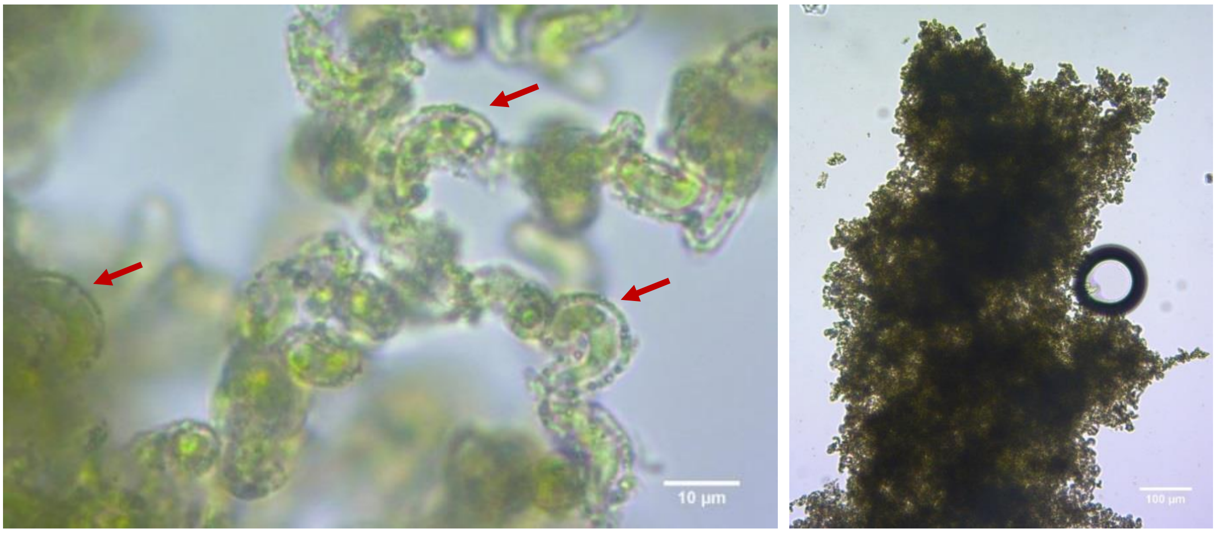


*Figure S11: Attachment of Eu-doped $TiO_2$ to algae revealing attachment to transparent, sheath-like structure around algal cells (red arrow). This phase-contrast image (left = 1000×, right = 100×) was captured after 3 h incubation with 100 mg/L.*

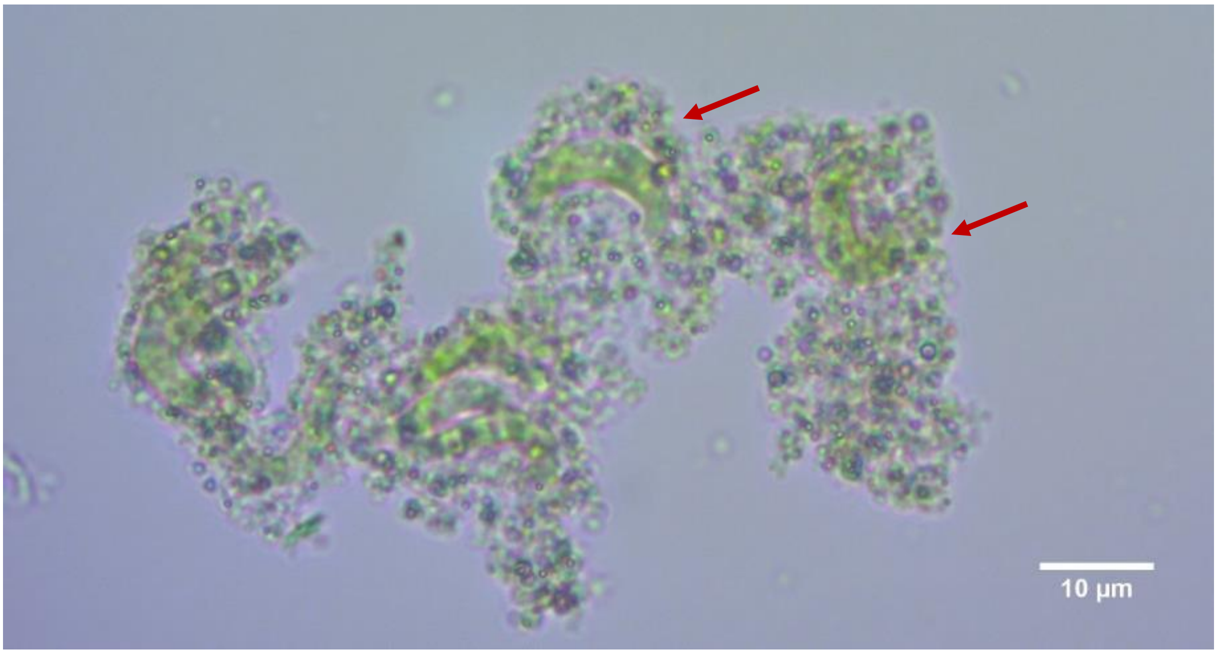


*Figure S12: Attachment of Eu-doped $TiO_2$ to algae, revealing particles spread over a small area with incorporated algae cells (red arrow). This phase-contrast image (1000× magnification) was captured after 3 h incubation with 100 mg/L Eu-doped $TiO_2$.*

# 7 Attachment of Fe-doped $TiO_2$ to the green algae *Raphidocelis subcapitata* – short-term test

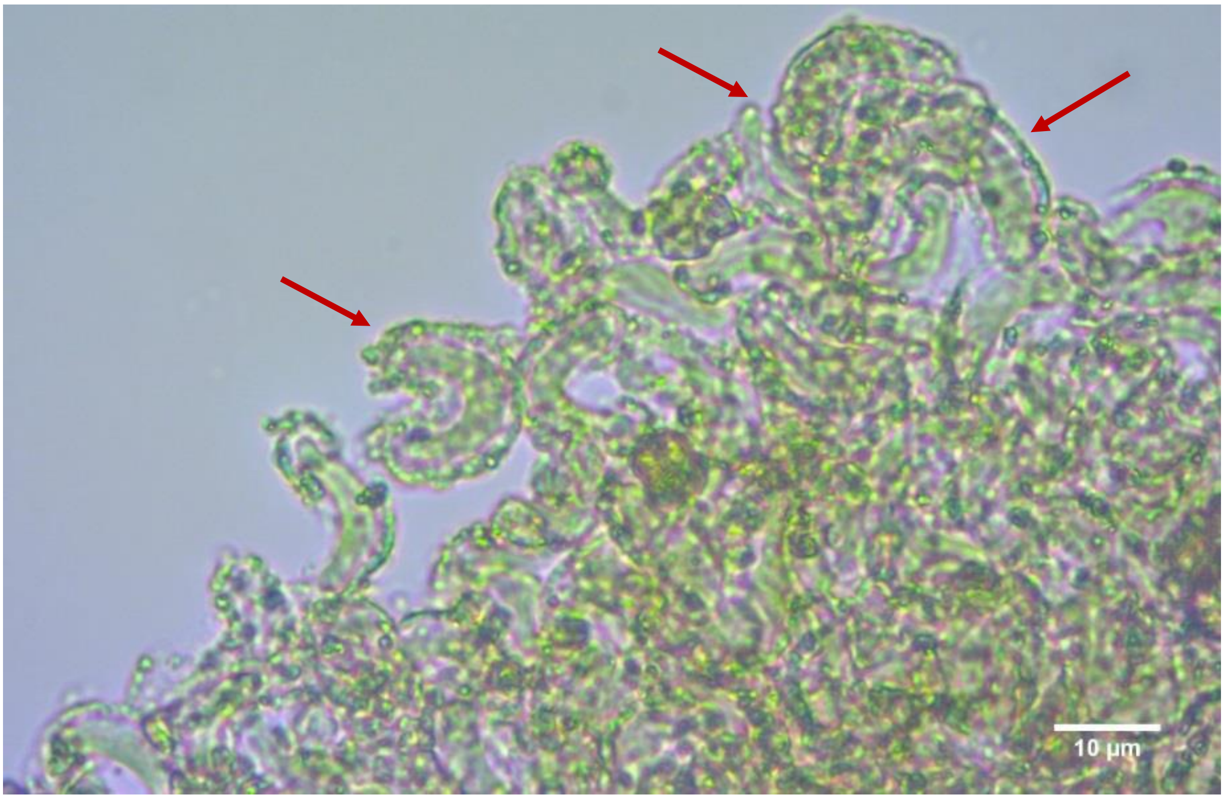


*Figure S13: Attachment of Fe-doped $TiO_2$ to algae (red arrow). This phase-contrast image (1000× magnification) was captured after 3 h incubation with 100 mg/L Fe-doped $TiO_2$..*

# 8 Attachment of $TiO_2$ NM-105 to the green algae *Raphidocelis subcapitata* – short-term test

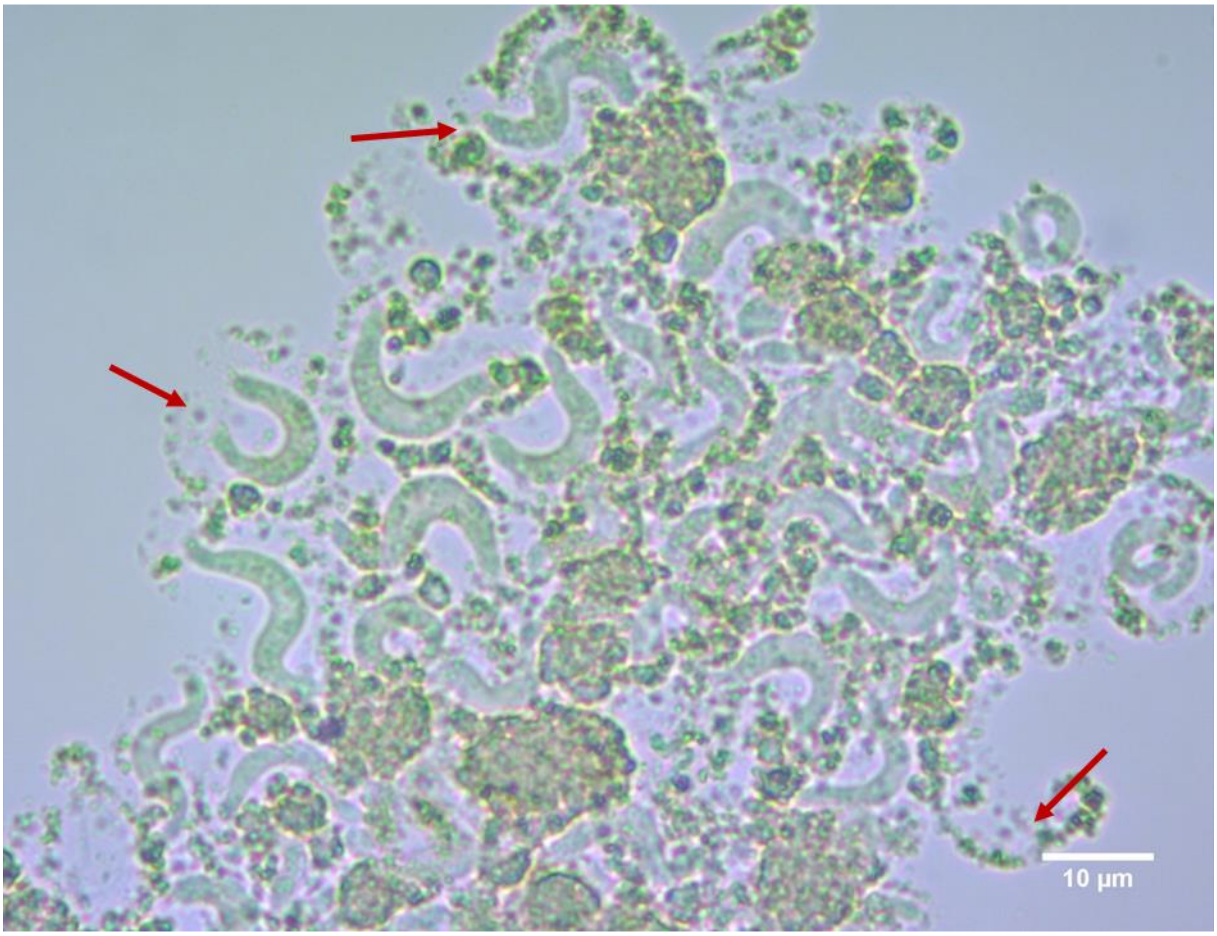


*Figure S14: Attachment of $TiO_2$ NM-105 to algae, showing nanoparticle attachment to algal cells and transparent, algae-shaped structures (red arrow). This phase-contrast image (1000× magnification) was captured after 3 h incubation with 100 mg/L $TiO_2$ NM-105.*

# 9 Attachment of $TiO_2$ NM-104 to the green algae *Raphidocelis subcapitata* – short-term test

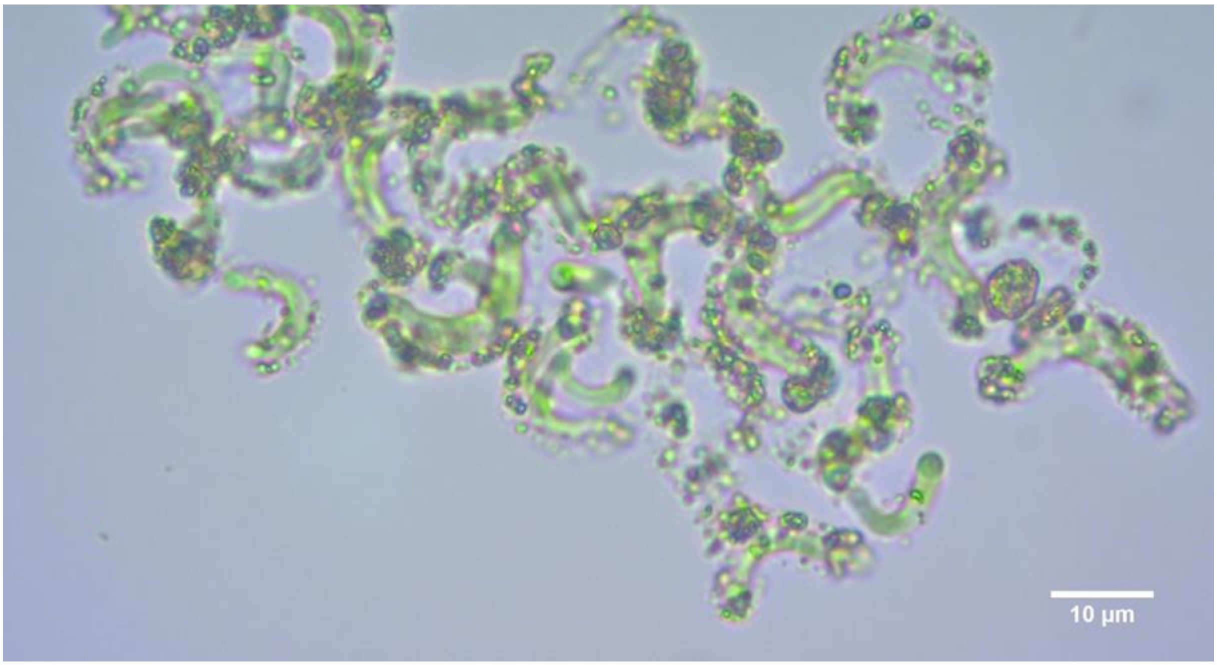


*Figure S15: Attachment of $TiO_2$ NM-104 to algae, showing loose formation of smaller agglomerates. This phase-contrast image (1000× magnification) was captured after 3 h incubation with 100 mg/L $TiO_2$ NM-104.*

# 10 Attachment of Eu-doped $TiO_2$ to the green algae *Raphidocelis subcapitata* – growth inhibition test

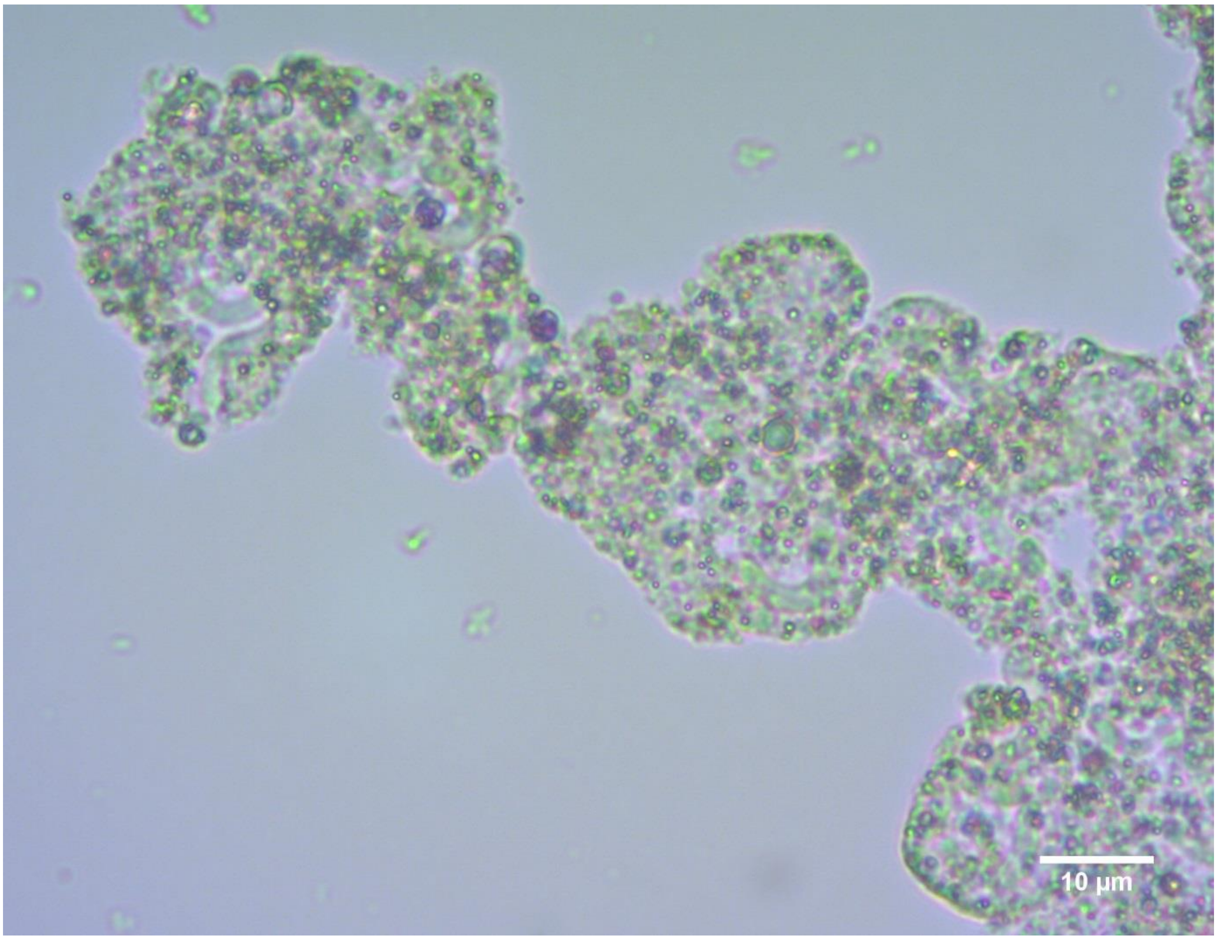


*Figure S16: Attachment of Eu-doped $TiO_2$ to algae. This phase-contrast image (1000× magnification) was captured during a 72-h growth inhibition test with 2 mg/L Eu-doped $TiO_2$.*

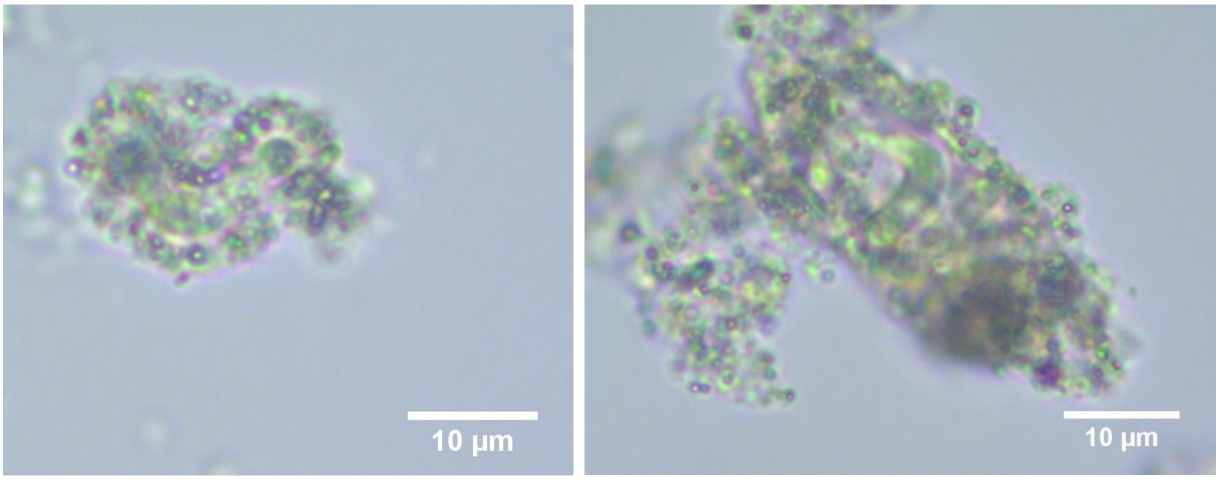


*Figure S17: Attachment of Eu-doped $TiO_2$ to algae. This phase-contrast image (1000× magnification) was captured during a 72-h growth inhibition test with 18 mg/L Eu-doped $TiO_2$.*

## 1 1 Attachment of $TiO_2$ NM-104 to the green algae *Raphidocelis subcapitata* – growth inhibition test

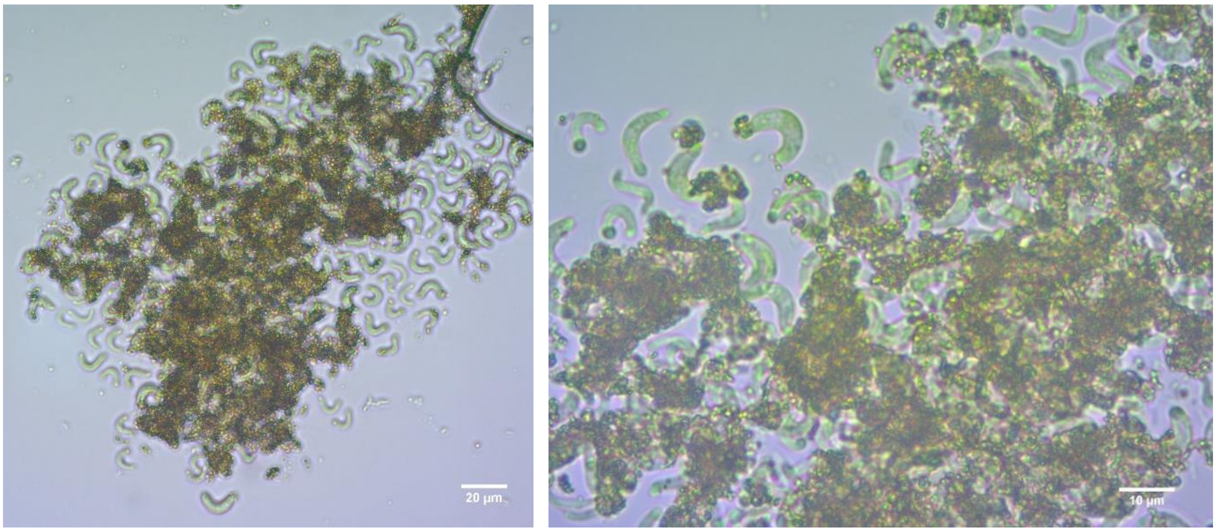


*Figure S18: Attachment of $TiO_2$ NM-104 to algae. This phase-contrast image (left = 400×, right = 1000×) was captured during a 72-h growth inhibition test with 7.5 mg/L $TiO_2$ NM-104.*

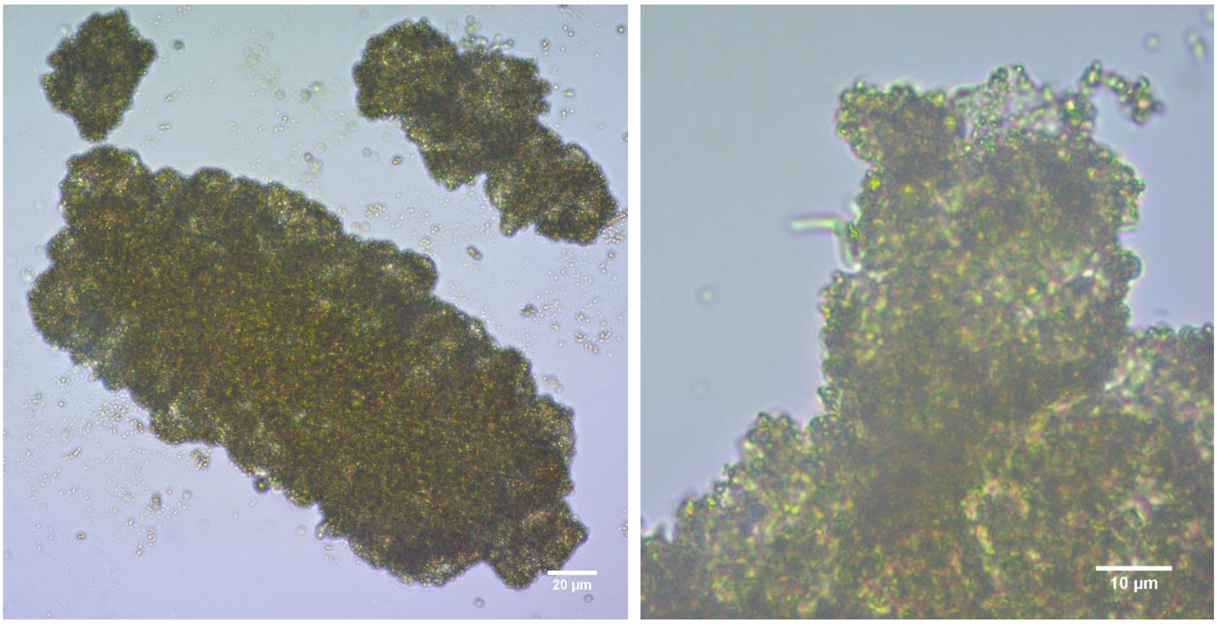


*Figure S19: Attachment of $TiO_2$ NM-104 to algae. This phase-contrast image (left = 400×, right = 1000×) was captured during a 72-h growth inhibition test with 30 mg/L $TiO_2$ NM-104.*

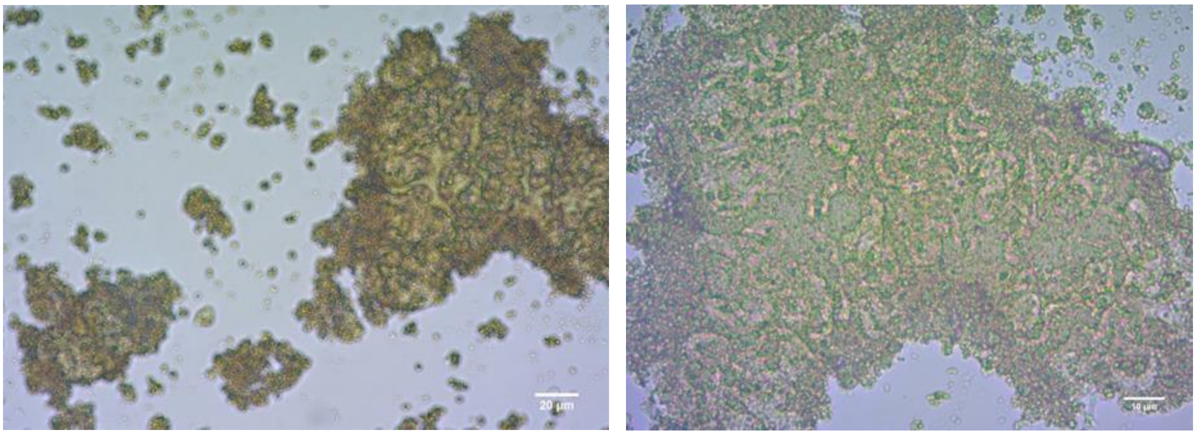


*Figure S20: Attachment of $TiO_2$ NM-104 to algae. This phase-contrast image (left = 400x, right = 1000x) was captured during a 72-h growth inhibition test with 120.0 mg/L $TiO_2$ NM-104.*
